# Crucial role of telomere maintenance-related genes in survival prediction and subtype identification in colorectal cancer

**DOI:** 10.3389/fmolb.2026.1728178

**Published:** 2026-08-28

**Authors:** Feng Huang, Jung-Yin Fong, Chin Tat Ng, Karthikkumar Venkatachalam, Mohammed Abdullah Alshawsh, Chun-Wai Mai, Yik-Ling Chew, Malarvili Selvaraja

**Affiliations:** 1 Faculty of Pharmaceutical Sciences, UCSI University, Kuala Lumpur, Malaysia; 2 Faculty of Applied Sciences, UCSI University, Kuala Lumpur, Malaysia; 3 Faculty of Medicine, University Kebangsaan Malaysia, Kuala Lumpur, Malaysia; 4 Centre for Cancer Prevention and Drug Development, Department of Internal Medicine, Hematology-Oncology Section, University of Oklahoma Health Sciences Centre, Oklahoma City, OK, United States; 5 Faculty of Medicine, Nursing and Health Sciences, Monash University, Clayton, VIC, Australia; 6 Department of Pharmacology, Faculty of Medicine, Universiti Malaya, Kuala Lumpur, Malaysia; 7 Centre for Cancer and Stem Cell Research, Institute of Research, Development and Innovation (IRDI) IMU University, Kuala Lumpur, Malaysia; 8 Department of Structural and Cellular Biology, Tulane University School of Medicine, New Orleans, LA, United States

**Keywords:** colorectal cancer, drug sensitivity, immune infiltration, PDE1B, prognostic model, telomere maintenance-related genes

## Abstract

**Background:**

Telomere maintenance-related genes (TMRGs) are implicated in Colorectal cancer (CRC) development, but their prognostic value and clinical relevance remain insufficiently explored. This study aims to develop a TMRG-based prognostic model and elucidate its clinical utility in CRC management.

**Methods:**

The Cancer Genome Atlas database was utilized to download RNA-seq data from 638 CRC and 51 control samples. Differential expressed genes were screened and intersected with 2086 TMRGs, resulting in the identification of 976 TMRGs. Through univariate and multivariate Cox regression analysis, a prognostic model comprising three telomere maintenance-related biomarkers (*PDE1B, TFAP2B, and HSPA1A*) was developed and validated using an external dataset. By integrating the model risk score with clinical features, a nomogram was constructed to predict the survival outcomes of CRC patients. Additionally, an in-depth investigation of the immuno-infiltration, functional variation and drug sensitivity analysis were performed in two risk subgroups defined by the prognostic model. Finally, the functional significance of *PDE1B* in CRC cell lines was investigated through MTT assays, cell colony formation assays, transwell assays and flow cytometry.

**Results:**

A total of 976 DE-TMRGs were enriched in telomere/DNA replication pathways. A three-gene signature (PDE1B, TFAP2B, and HSPA1A) stratified patients into high- and low-risk groups with divergent survival (AUC >0.60, validated externally). High-risk patients had advanced N/M stages, elevated M0/M2 macrophages, reduced CD4^+^ memory T cells, and upregulated immune checkpoints. Nomogram integrating risk score, age, and N/M stage accurately predicted 1-/3-/5-year survival. Low-risk patients showed greater 5-fluorouracil sensitivity. *PDE1B* expression was significantly reduced in CRC tissues and correlated with advanced stages. Functional assays confirmed PDE1B overexpression suppressed proliferation, migration, invasion, and induced apoptosis in CRC cells.

**Conclusion:**

This study identifies a moderately predictive telomere maintenance-related gene signature as an independent prognostic predictor in CRC. The risk stratification model effectively discriminates patients with distinct survival patterns, tumor microenvironments, and therapeutic responses, while the integrated nomogram offers additional reference information for survival analysis, albeit with only moderate predictive accuracy. These findings indicate telomere maintenance-related gene signature could serve as a preliminary auxiliary risk stratification tool for postoperative CRC patients, *PDE1B* may also serve as a potential epithelial tumor-suppressor target for future preclinical studies.

## 1 Introduction

Colorectal cancer (CRC) remains a leading cause of cancer-related mortality worldwide, ranking third in incidence among malignancies (Dekker et al., 2019; Schreuders et al., 2015; Ma et al., 2020). Despite advancements in early detection and treatment, the 5-year survival rate for advanced-stage patients remains low (12–19%) (Dekker et al., 2019). Current clinical biomarkers, such as carcinoembryonic antigen (CEA) and carbohydrate antigen 199 (CA199), exhibit limited specificity and sensitivity, underscoring the critical need for improved CRC diagnostics and prognostics (Lakemeyer et al., 2021; Beniwal et al., 2023). A Substantial efforts have been devoted to identifying molecular predictors for CRC; however, no biomarker-based stratification tool has yet been successfully integrated into routine clinical decision-making, largely owing to the complex interplay between tumor heterogeneity and the immune microenvironment (Hanahan and Weinberg, 2011).

Telomeres are specialized structures at the ends of eukaryotic linear chromosomes, consisting of chromosomal telomeric regions and telomere binding proteins (Shay and Wright, 2019; de Lange, 2009; Moyzis et al., 1988). It is now widely accepted that the primary function of telomeres is to safeguard chromosome ends and maintain genomic stability (Blackburn et al., 2015). During normal cell division, telomeres progressively shorten, ultimately inducing cellular senescence or apoptosis. However, when cells acquire mutations, such as inactivation of tumor suppressor genes like *p53* or *Rb* (Okamoto and Seimiya, 2019, Hastie et al., 1990, Henson et al., 2002; Nassour and Karlseder, 2026), that enable them to bypass senescence, excessive telomere shortening can result in chromosomal instability, a condition referred to as telomere crisis (Willeit et al., 2010). This instability may give rise to genomic aberrations, including chromosomal fusions and breaks, which contribute to the initiation and progression of carcinogenesis (Bloom and Karlseder, 2025).

Recent CRC studies have supported the prognostic and therapeutic relevance of telomere maintenance genes, but their cellular origin and functional contribution remain incompletely resolved (Wang et al., 2025; Ma et al., 2026). Two independent studies published in 2025 have pioneered the prognostic utility of TMGs in CRC. Wang et al. developed a seven-gene risk model (*CDC25C, CXCL1, RTL8C, FABP4, ITLN1, MUC12,* and *ERI1*) and identified two CRC subtypes with distinct clinical outcomes (Wang et al., 2025). Concurrently, Ma et al. constructed a two-gene prognostic signature (*CDC25C* and *USP39*) and validated its predictive performance across multiple cohorts (Ma et al., 2026). While these studies have firmly established the prognostic relevance of TMGs in CRC, they converge on *CDC25C*, a cell cycle regulator, as a common component, suggesting that current signatures may preferentially capture cell cycle-related pathways rather than the full spectrum of telomere biology, including immune modulation and microenvironment remodeling. Notably, functional validation in these studies was limited to a single gene (*MUC12* in Wang et al.; none in Ma et al.), leaving the biological roles of most candidate genes in CRC unexplored.

In this study, we show that a three-gene telomere maintenance-related signature, centered on *PDE1B*, effectively stratifies CRC patients into high- and low-risk groups with markedly different survival outcomes, and that this stratification uncovers differential immune vulnerabilities and chemotherapeutic sensitivities. Through comprehensive analysis of TCGA-CRC RNA-seq data (638 tumors, 51 normals) with external validation in GSE28722 (121 cases), we identified 976 differentially expressed TMRGs enriched in telomere maintenance and DNA replication pathways. A parsimonious Cox regression model comprising *PDE1B, TFAP2B, and HSPA1A* was constructed and validated. High-risk patients exhibited advanced pathological N/M stages, heightened infiltration of immunosuppressive M0/M2 macrophages, reduced CD4^+^ memory T cells, and elevated expression of 34 immune checkpoints providing a detailed characterization of an immune-excluded, dysfunctional tumor microenvironment. A nomogram integrating risk scores with age and pathologic N/M stage accurately predicted 1-, 3-, and 5-year overall survival. Importantly, drug sensitivity analysis identified enhanced 5-fluorouracil efficacy in the low-risk group, providing a potentially actionable therapeutic indication.

Collectively, this study establishes a TMRG-centered framework that bridges molecular profiling with clinical decision-making in CRC. We anticipate that this prognostic framework, coupled with the companion nomogram, will provide a potentially clinically applicable tool for risk-adapted patient management, and that *PDE1B* may represent a new avenue for therapeutic intervention in CRC.

## 2 Materials and methods

### 2.1 Data acquisition and preprocessing

The TCGA-CRC was derived from the UCSC Cancer Genomics Browser (https://genome.ucsc.edu/) and comprised RNA-seq data from 638 CRC patients, including 606 samples with survival information, as well as 51 control samples, which were used as the training set. The external validation data came from the GSE28722 dataset was sourced from the GEO database (http://www.ncbi.nlm.nih.gov/geo). The GSE28722 dataset (platform GPL13425) is a microarray-based dataset that includes gene expression profiles and survival information from 121 CRC samples, and it was utilized as an independent external validation set to assess the robustness of our model across different technical platforms. Then, 2,086 telomere maintenance-related genes were obtained from previous reports (Li et al., 2022). For the TCGA-CRC training cohort, we used the raw count and FPKM data as downloaded from the UCSC Cancer Genomics Browser without additional normalization. For the GSE28722 validation cohort, we directly used the normalized expression matrix provided by the original authors. The study ensure that all our methods are implemented and designed in strict accordance with the operating procedures provided by the public data provider. Adhering to these guidelines is essential to ensure the integrity and reliability of our work. Additionally, all datasets utilized in this study are publicly accessible online.

To ensure the reliability of our downstream analyses, we systematically evaluated potential batch effects within the TCGA-CRC (COAD/READ) RNA-seq dataset. We performed Principal Component Analysis (PCA) on the normalized expression matrix and examined sample clustering with respect to key metadata variables, including tissue source site (TSS) and sequencing platform. The PCA plot did not reveal any obvious separation or clustering of samples based on these technical factors (S6 Figure 6). To quantitatively assess the presence of batch effects, we calculated the Dispersion Separability Criterion (DSC) index. The DSC value was 0.9995 with a corresponding *p*-value of 0.58 (*p* > 0.05), which indicates that there is no statistically significant batch effect in our dataset. Therefore, no batch correction was applied to the data used in this study.

### 2.2 Identification of differentially expressed genes (DEGs) in CRC

Prior to differential expression analysis, lowly expressed genes were filtered out, resulting in a final set of 44,688 genes for analysis. DEGs between the CRC and control groups were identified using the DESeq2 package (v 1.34.0) (Chen et al., 2024) in the TCGA-CRC dataset with adjusted *P* value <0.05 and |log 2 FC| > 0.5. The results of the differential analysis were illustrated using a volcano plot generated by the ggplot2 package (v 3.3.5) (Zhao et al., 2021). Additionally, a heatmap was used to show the expression of the top 10 most significantly up- and downregulated genes.

### 2.3 Screening and functional enrichment of differentially expressed TMRGs (DE-TMRGs)

DE-TMRGs were screened by overlapping DEGs and TMRGs. To gain an initial, global perspective on the biological processes and pathways collectively engaged by these CRC-dysregulated telomere-related genes, we performed Gene Ontology (GO) and Kyoto Encyclopedia of Genes and Genomes (KEGG) enrichment analyses on the DE-TMRGs using the “clusterProfiler” package (v 4.2.2) (Li et al., 2022). For GO analysis, the background (universe) comprised all human genes with GO annotations in the org.Hs.eg.db database; for KEGG analysis, the background comprised all human genes with KEGG pathway annotations. Enriched terms with an adjusted *P* value <0.05 (Benjamini–Hochberg method) were considered significant. It should be noted that this analysis is descriptive in nature and serves to characterize the pre-selected DE-TMRG gene set, rather than to test whether telomere maintenance pathways are globally overrepresented among all DEGs in CRC.

### 2.4 Prognostic model construction and validation

The univariate Cox algorithm (Ye et al., 2021) was conducted for DE-TMRGs to acquire candidate genes. Afterwards, multivariate Cox algorithm was performed on the basis of candidate genes. Then, the genes gained were utilized as biomarkers for this study. The risk score formula of the Cox model is: Risk score = $\sum_{1}^{n}\text{coef}\left(\text{genei}\right)*expression\left(\text{genei}\right)$ . A positive beta coefficient indicates that elevated gene expression increases the risk of patient death (high expression, high risk), while a negative beta coefficient indicates that elevated gene expression reduces the risk of death (high expression, low risk); the prognostic value of genes in the model is determined by both coefficient direction and HR value. The survival ROC package (v 1.0.3) (In and Lee, 2019) was utilized to compute area under the curve (AUC) values for receiver operating characteristic (ROC) curves to assess the predictive accuracy of the model, and the Kaplan-Meier (K-M) survival curves were drawn. The risk model was verified with GSE28722 dataset.

### 2.5 Clinical features and survival analysis

Firstly, the clinical information (age, pathologic-N, gender, pathologic-T, and pathologic-M) was analyzed for differences between two risk subtypes. Then, survival analysis was performed using the survival package (v 3.3-1) (Mosmann, 1983) for different clinical characteristics in two risk subgroups, and K-M curves were plotted to present the results.

### 2.6 Nomogram construction and independent prognostic analysis

Independent prognostic analyses were performed on clinical characteristics and risk score in the TCGA-CRC dataset. Immediately after, the nomogram was constructed and visualized via rms package (v 6.2.0). Next, a calibration curve was plotted to judge the model performance.

### 2.7 Pathway enrichment based on gene set enrichment analysis (GSEA) and gene set variation analysis (GSVA)

In order to understand the functional differences between the two risk subgroups, the GSVA package (v 1.42.0) (Hänzelmann et al., 2013) was utilized to compute ssGSEA scores for all entries or pathways in the GO and KEGG databases in two groups. Then, the top 10 up- and downregulated pathways in each database were demonstrated with heatmaps. To further investigate the potential functions of prognostic genes, a GSEA analysis was performed. First, the Spearman correlation coefficients between each gene and the entire gene set were calculated; the genes were then sorted by these coefficients and input into GSEA as a gene list. Enrichment analysis was conducted using the GSEA function in the clusterProfiler package (v 4.14.0), with the TERM2GENE parameter set to call the KEGG and GO gene sets, respectively. set pAdjustMethod to BH and pvalueCutoff to 1 to retain all results, then extract significant entries using the filter criteria |normalized enrichment score (NES)| > 1 and adjusted *P* value <0.05.

### 2.8 Drug sensitivity analysis

The IC_50_ values of the chemotherapy drugs were computed using the once Predict package and then the differential drug sensitivity between the two risk subgroups was compared (*P* < 0.05). Next, the box plot was drawn to show the results for 5-fluorouracil and the ten drugs with the lowest *P* values.

### 2.9 Immune microenvironment analyses

The stromal score and immune score were computed for the two risk subtypes using the estimate package (v 1.0.13) (*P* < 0.05). Then, the proportion of 22 immune cell subtypes was computed for each sample by the CIBERSORT algorithm (Newman et al., 2015). Subsequently, the infiltration of the remaining immune cells between the two risk subgroups was analyzed after excluding the immune cells with an infiltration value of 0 in 75% of the samples. The results of the analysis were presented by the proportion and violin diagrams. Afterwards, the correlation coefficients between differential immune cells were computed by the stats package (v 4.1.0), and the results were visualized using network plot. In addition, the expression of 48 immune checkpoints was assessed for differences between the two risk subgroups. Meanwhile, tumor immune dysfunction and exclusion (TIDE) Dysfunction, and Exclusion scores were computed between the two risk subgroups to assess the difference in sensitivity to immunotherapy between the two groups. To clarify the correlation between the 3 scores and the risk score, we calculated the correlation between them using the stats package (v 4.1.0). The Immune Prognostic Score (IPS) is an indicator to assess the immune status and prognosis of patients with tumor. In this study, differences in scores between the two risk subgroups were analyzed using CRC IPS data downloaded from the IPS website, and the results were presented by box plots.

### 2.10 Expression analysis of the three prognostic biomarkers

The chromosomal localization analysis of biomarkers was performed by the RCircos package (v 1.2.2). Finally, the expression levels of biomarkers between control and CRC groups were compared in the training set.

### 2.11 The function analysis of *PDE1B* in CRC

The UALCAN database (https://ualcan.path.uab.edu/) was utilized to analyze the expression of PDE1B in colorectal cancer patients.

### 2.12 Cell culture

HCT116 and SW480 human colorectal cancer cell lines were obtained from American Type Culture Collection (ATCC, Manassas, VA, United States) and were cultured in Dulbecco’s Modified Eagle’s Medium and Leibovitz medium, respectively, supplemented with 10% fetal bovine serum, and 1% penicillin and streptomycin. All cells were incubated in a humidified atmosphere of 5% CO_2_ at 37 °C.

### 2.13 PDE1B overexpression

The full-length coding sequence of the *PDE1B* gene was cloned into the LV18 lentiviral vector purchased at GENECHEM (Shanghai, China), and LV18 was used as a negative/vector control. CRC cells were infected with LV18-DLX5 or LV18 according to the manufacturer’s instructions. Infection efficiency was assessed by qRT-PCR. Puromycin (Beyotime Biotechnology, China) was then used to select and establish stably expressing cell lines.

### 2.14 Quantitative real-time PCR (RT-qPCR)

Total RNA was extracted from HCT116 or SW480 cells by VAMNE Magnetic Cell/Tissue Total RNA Kit (Prepackaged for RMA101-C2-P3) (Vazyme, China) according to the manufacturer’s instructions. Approximately 5 × 10^6^ fresh cells were used in each assay. The extracted total RNA was quantifiable by a Nanodrop 2000 spectrophotometer (Thermo Fisher Scientific, United States) with acceptable purity. Reverse transcription was performed using a reverse transcription kit (HyperScript™ RT SuperMix for qPCR). qRT-PCR was performed with Hieff qPCR SYBR Green Master Mix (Yeasen, 11202ES08) on Applied Biosystems 7500 Fast Real Time System (ABI, 4,351,107). Relative mRNA levels were determined using the ^ΔΔ^Ct method. The change fold of the negative control (NC) in each experiment was set to 1, and the outcomes of the three experiments were subjected to statistical analysis. One-way ANOVA was employed to compare differences among many groups, while t-tests (and nonparametric tests) were utilized to assess differences between two groups. Analysis was performed using GraphPad Prism software. GAPDH was reference control for normalization (Yang et al., 2024). All experiments were performed in triplicate. Information on the primers is summarized in the Table 1.

**TABLE 1:** Sequences of primers used in this study.

Gene	Sequences
*PDE1B*	Forward: 5′⁃GGAAGGGCTGGAATAGGATAG⁃3′
	Reverse: 5′⁃AGAAAGGGAGGGAAGGAAGA⁃3′
*GAPDH*	Forward: 5′⁃TCGGAGTCAACGGATTTGGT⁃3′
	Reverse: 5′⁃TTCCCGTTCTCAGCCTTGAC⁃3′

### 2.15 MTT assay

HCT116 and SW480 cells were seeded at a density of 3 × 10^3^ cells per well in 96-well plates and cultured for 24 h. At each detection point, 10 μL MTT reaction solution (298-93-1, Solarbio, China) was added to each well. The plates were then incubated at 37 °C for 2 h. After removing the supernatant, 100 μL of DMSO was added to each well to dissolve the formazan crystals. The absorbance at 570 nm was measured by a microplate reader (BMG Spectrostar, BMG Labtech, Germany). All experiments were performed in triplicate. The results were analyzed by Prism 9.0 (GraphPad, La Jolla, CA, United States).

### 2.16 Cell colony formation assay

Approximately 600 cells were seeded in each well of six-well plates and cultured at 37 °C in a humidified atmosphere with 5% CO_2_. The medium was refreshed every 72 h. After 14 days, the cells were fixed with 4% paraformaldehyde for 15 min and stained with 0.1% crystalline violet staining solution (G1062, Solarbio, China) for 15 min. Colonies were subsequently photographed and quantified using ImageJ software. All experiments were performed in triplicate (Schneider et al., 2012).

### 2.17 Invasion and migration assays

For the invasion assay, transwell upper chambers were precoated with Matrigel (C0372, Beyotime, China). In the 8-μm pore chamber (353097, BD Falcon, United States) coated with Matrigel containing serum free medium, equal numbers of cells (5 × 10^4^/well) were plated. As an attractant, complete culture media was added to the bottom chamber. Forty-eight hours later, cells in the upper chamber were wiped off. Migrated and invaded cells on the lower membrane surface were fixed, stained with crystal violet, and photographed. ImageJ software was utilized to count the number of migrated and invaded cells. Migration assay was carried out the same as invasion assay, but without the utilization of Matrigel.

### 2.18 Cell apoptosis analysis

The Annexin V-FITC Apoptosis Detection Kit (E-CK-A211, Elabscience, China) was used to perform apoptosis analysis following the manufacturer’s instructions. HCT116 and SW480 cells from the different treatment groups were trypsinized, collected by centrifugation at 1,000 rpm for 5 min, and then washed with cold 1× PBS. Subsequently, the cells were incubated with propidium iodide (PI) and annexin V reagents in the dark at room temperature for 15 min. Flow cytometric analysis was performed using a BD FACSAria IIIu instrument, and apoptotic rates were quantified using FlowJo software (version 10.10, United States).

### 2.19 Statistical analysis

All bioinformatics analyses were undertaken in R software (version 4.0.2). All experiments and analyses were performed in triplicate to ensure accuracy and reliability. Numerical data are displayed as the mean ± standard deviation (SD). All statistical analyses were carried out with GraphPad Prism 9.0 (GraphPad, La Jolla, United States). The statistical significance of the differences was analyzed by Student’s t test, unpaired t-test, and one-way ANOVA according to the test of homogeneity of variances. Statistical significance was set as follow: *, P < 0.05; **, P < 0.01; and ***, P < 0.001.

## 3 Results

### 3.1 DE-TMRG identification and functional enrichment

**FIGURE 1:**
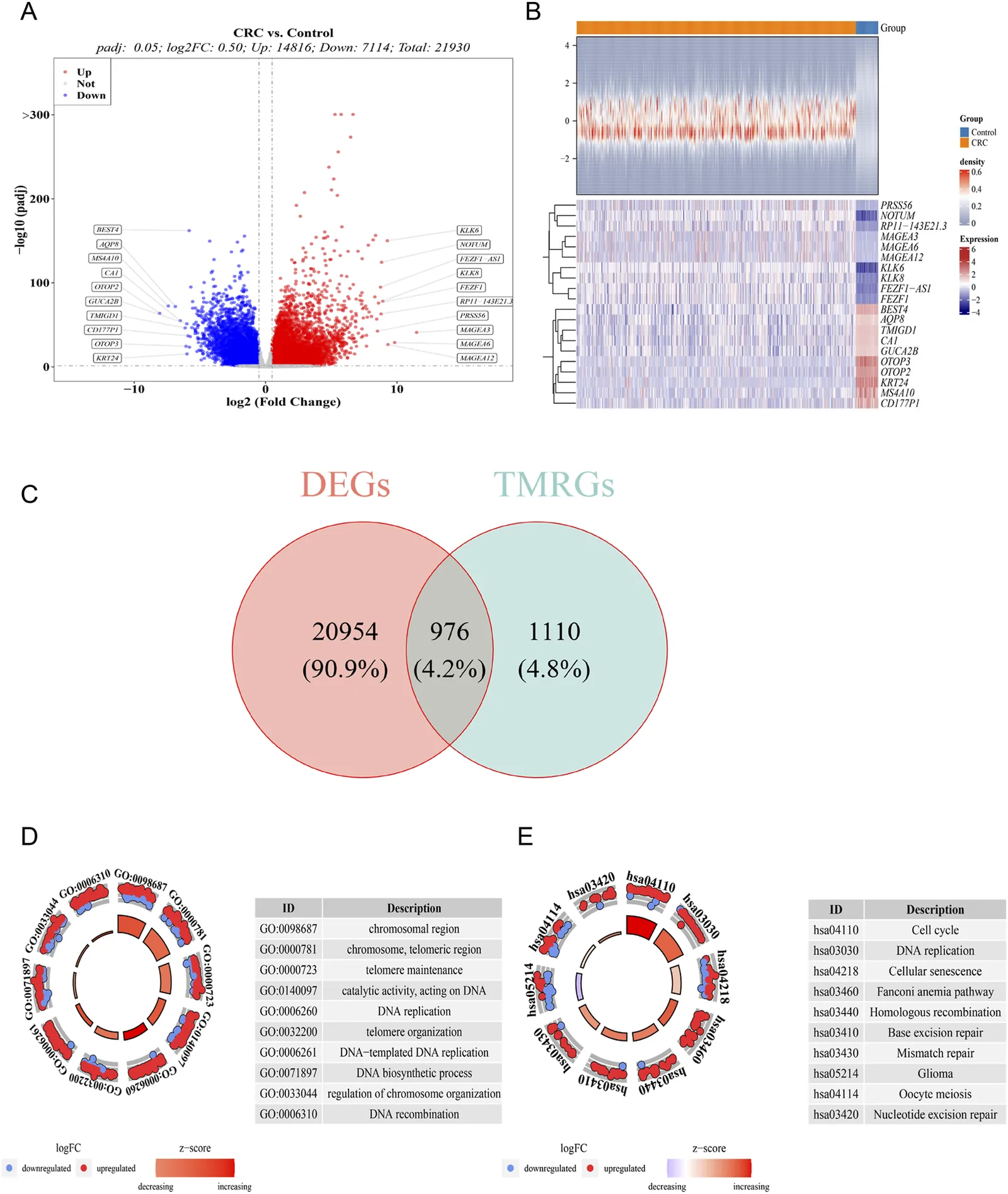
Identification and functional enrichment of DE-TMRGs. **(A)** Volcano plot of DEGs; **(B)** Heatmap of DEGs. There are two parts to the heatmap: the upper portion shows the expression density of differential genes, while the lower portion displays the top 10 genes among the up- and down-regulated DEGs. **(C)** Venn diagram of 976 DE-TMRGs. DEGs were differentially expressed genes and TMRGs are telomere maintenance-related genes. **(D)** GO analysis of DE-TMRGs; **(E)** KEGG analysis of DE-TMRGs.

Based on the TCGA database, a total of 21,930 differentially expressed genes were identified between the CRC and control groups, comprising 14,816 up-regulated genes and 7,114 down-regulated genes (Figure 1A; Supplementary Table S1). This large number of DEGs is attributed to the inclusion of non-coding RNA transcripts in the analysis and the relatively lenient but commonly used thresholds (|log2FC| > 0.5, FDR <0.05) for capturing subtle but biologically relevant expression changes in a large cohort. The density plot revealed that most of the DEGs were expressed between −2 and 2, with fewer genes near 0 (Figure 1B). Subsequently, 976 DE-TMRGs were screened by overlapping 21,930 DEGs and 2,086 TMRGs (Figure 1C). Enrichment analysis results indicated that the DE-TMRGs implicated 736 GO-BP entries, 118 GO-CC entries, 158 GO-MF entries, and 66 KEGG pathways. As expected, given their definition as telomere maintenance-related genes, GO term annotation showed that DE-TMRGs were primarily associated with “chromosome, telomeric region,” and “telomere maintenance” (Figure 1D; Supplementary Table S2). KEGG enrichment analysis highlighted pathways such as “cell cycle” and “DNA replication” (Figure 1E; Supplementary Table S3). This result provides a data-driven characterization and a conceptual link between our identified DE-TMRG set and core telomere-associated and DNA metabolic functions, serving as a preliminary overview of this specific functional subset of dysregulated genes in CRC.

### 3.2 Biomarkers screening and construction of a risk prediction model

**FIGURE 2:**
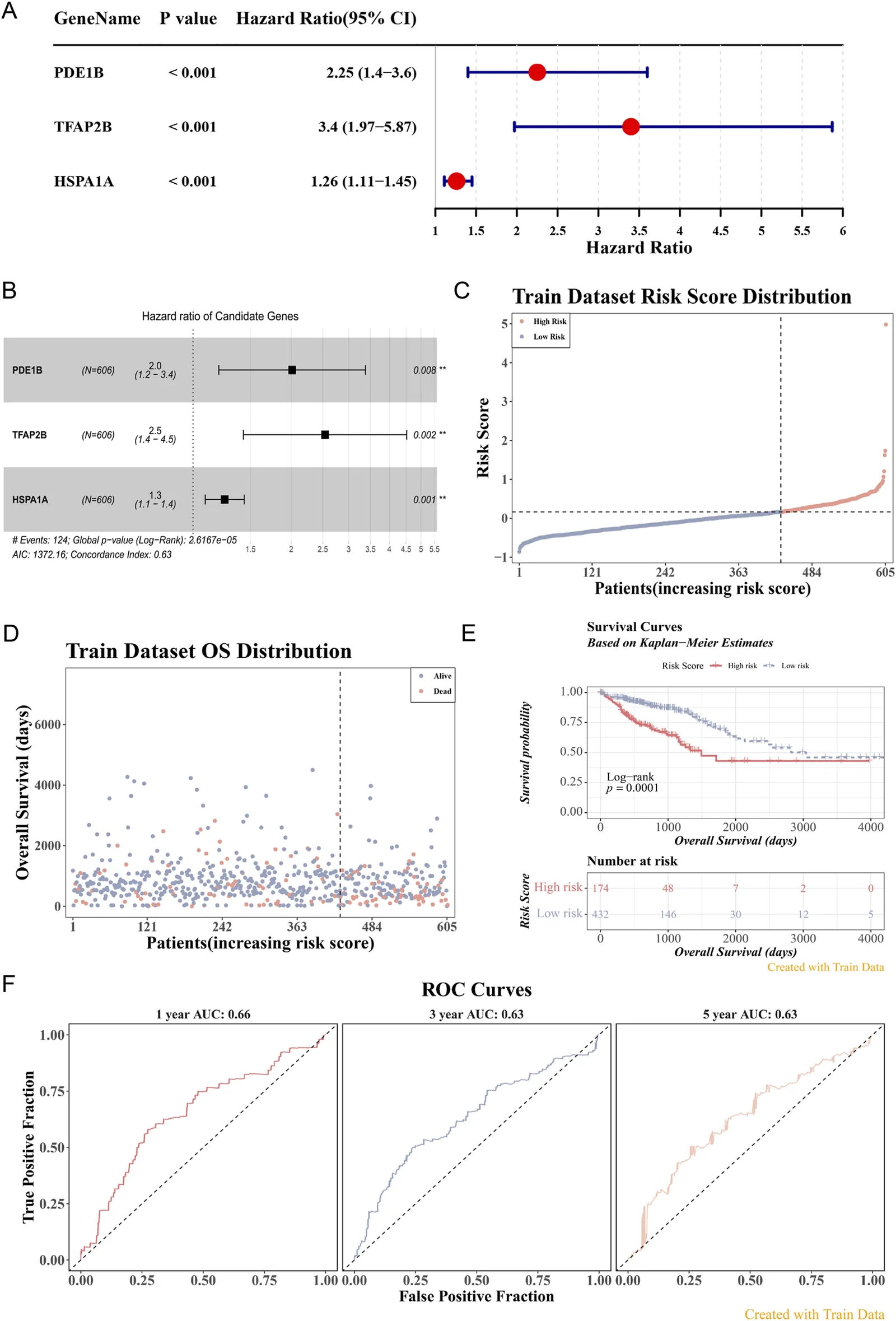
Screening of biomarkers and construction of risk model. **(A,B)** Univariate and multivariate Cox regression analyses for identifying prognostic biomarkers. **(C)** Risk Score Distribution Chart. **(D)** Survival state distribution Chart. **(E)** Kaplan-Meier survival curve. The upper part shows survival time versus probability of survival, and the bottom part lists risks. **(F)** ROC curve.

In total, three telomere maintenance-related biomarkers (*PDE1B*, *TFAP2B*, and *HSPA1A*) were acquired by univariate and multivariate Cox algorithm (Figures 2A,B). Multivariate Cox regression validated all three genes as independent prognostic factors, with *TFAP2B* presenting a positive regression coefficient (coef = 0.9323, HR = 2.5404, *p* = 0.0015), *PDE1B* (coef = 0.6999, HR = 2.0135, *p* = 0.0080), and *HSPA1A* (coef = 0.2238, HR = 1.2509, *p* = 0.0014). The risk score of patients was computed, and the patients were classified into high-risk (n = 174) and low-risk groups (n = 432) depend on the basis of optimal thresholds (0.1644556) (Figures 2C,D). Survival analysis curves demonstrated that the high-risk group of the training set had a lower survival rate (Figure 2E). The results of the ROC curves revealed that the model exhibited favorable predictive performance, with AUCs greater than 0.60 for 1-, 3-, and 5-year survival (Figure 2F). To further assess the model’s discriminative ability, Harrell’s concordance index (C-index) was calculated, yielding a value of 0.63 in the TCGA-CRC training cohort.

**FIGURE 3:**
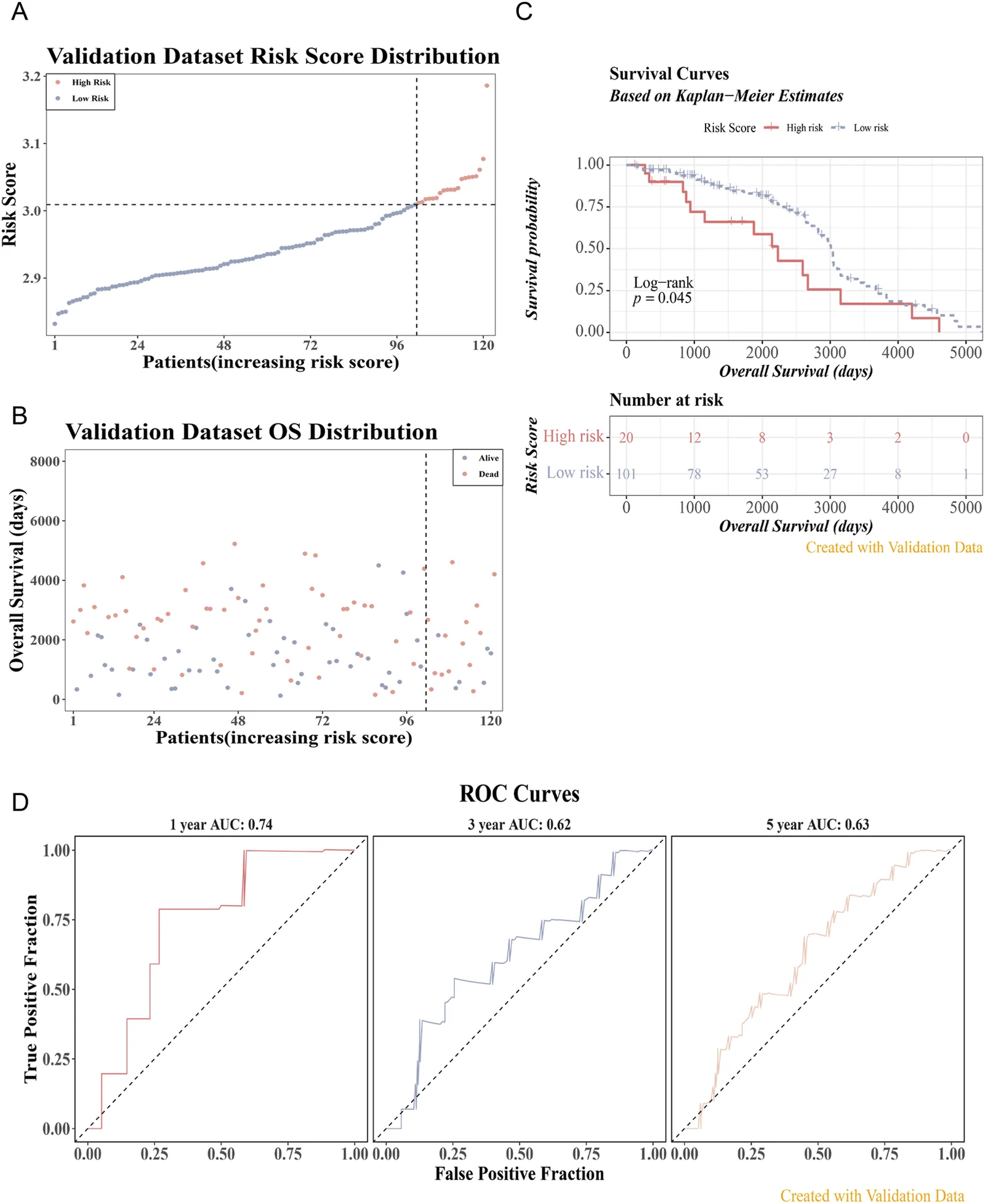
External validation of the model’s prognostic predictive ability. **(A)** Risk Score Distribution Chart. **(B)** Survival state distribution Chart. **(C)** K-M curve. **(D)** ROC curve.

Subsequently, we utilized the independent microarray-based validation set (GSE28722) to evaluate the predictive performance of the model. The risk profile plots and survival curves of the external validation set were consistent with those of the training set. Samples were sorted based on their risk scores and categorized into high and low-risk groups (Figure 3A). The distribution of survival status indicated that surviving samples were mostly concentrated in the region with lower risk scores and longer total survival time, whereas the deceased samples were predominantly clustered in the region with higher risk scores and shorter total survival time (Figure 3B). Further analysis displayed a significant difference (*p* = 0.045 < 0.05) in survival between the high and low-risk groups in the training set (Figure 3C). Additionally, The AUC values for the validation set exceeded 0.6 (Figure 3D). The C-index for the GSE28722 validation cohort was 0.57, indicating moderate but consistent performance across datasets. These findings are detailed in Supplementary Figure S7. The results indicated that the risk model can be helpful in predicting prognosis.

To investigate potential biological interplay among these three signature genes, we performed correlation and protein-protein interaction (PPI) network analyses. Pearson correlation analysis in the TCGA-CRC cohort revealed a significant positive correlation between the expression levels of *HSPA1A* and *TFAP2B* (r = 0.0596, p < 0.001), while *PDE1B* showed no significant correlation with either of the other two genes (Supplementary Figure S11). Furthermore, a PPI network constructed using the GeneMANIA database showed that *HSPA1A* and *TFAP2B* are connected through shared functional partners involved in transcriptional regulation and stress response pathways, but no direct physical interaction was found between any of the three proteins (Supplementary Figure S12). These findings suggest that while *HSPA1A* and *TFAP2B* may operate in related biological contexts, the three genes likely contribute to the prognostic power of our model through complementary, rather than directly interactive, mechanisms.

### 3.3 Clinicopathological features and survival analysis between two risk subtypes

**FIGURE 4:**
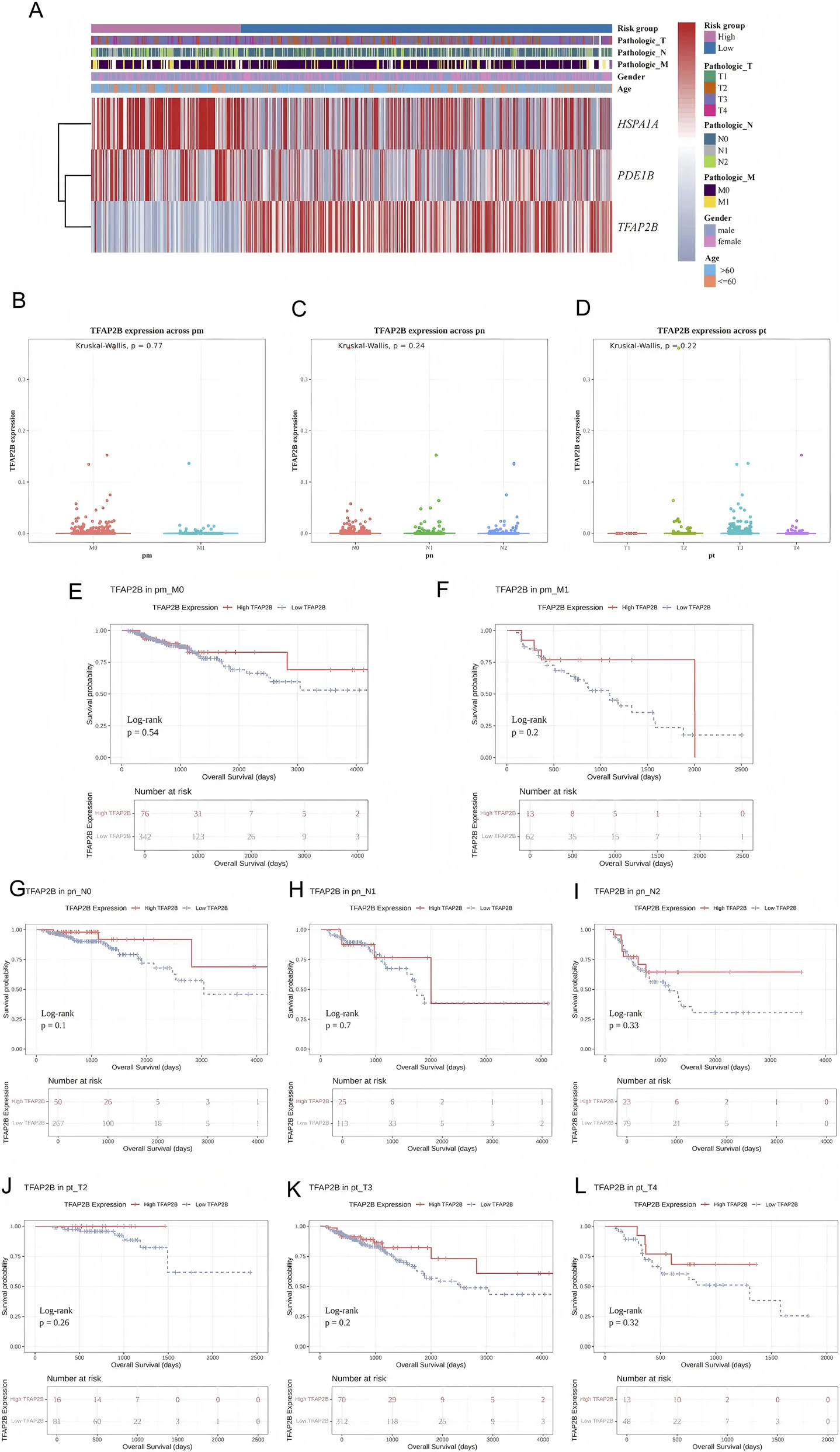
Clinical characterization and stratified survival analysis of TFAP2B in colorectal cancer. **(A)** Clinically relevant heatmap of three prognostic biomarkers (PDE1B, TFAP2B, HSPA1A) associated with pathological stages, gender and age. **(B–D)** Boxplots of TFAP2B expression across pathological M (pm), N (pn) and T (pt) stages (Kruskal–Wallis test: pm *p* = 0.77, pn *p* = 0.24, pt *p* = 0.22). **(E–L)** Kaplan-Meier survival curves of TFAP2B high vs. low expression in different pathological subgroups: **(E)** M0, **(F)** M1, **(G)** N0, **(H)** N1, **(I)** N2, **(J)** T2, **(K)** T3, **(L)** T4 (all log-rank p > 0.05).

To examine how risk scores are linked with patients’ clinical characteristics and survival, we analyzed the differences in risk scores between subgroups with varying clinical characteristics. Analysis of clinical characteristics revealed that risk scores were significantly different between pathologic (M, N and T) groups (Figure 4A). For *TFAP2B*, Kruskal–Wallis tests confirmed no significant expression differences across pathological T (*p* = 0.22), N (*p* = 0.24), or M (*p* = 0.77) stages (revised Figures 4B–D). Additional analyses of age and gender subgroups are provided in Supplementary Figure S1. The *p*-values for pathologic group M, N, T were (5.3e-05; 0.02; 0.0013), respectively, and not significantly different between age and gender groups (Supplementary Figure S1A). It indicates that patients are at increased risk as the stage of pathology advances. Stratified survival analysis of *TFAP2B* in T2-T4, N0-N2, and M0-M1 subgroups (revised Figures 4E–L) showed no significant overall survival differences (all log-rank *p* > 0.05), confirming that *TFAP2B* has no stable single-gene prognostic value and its prognostic effect relies on the synergistic action of the multi-gene signature.

### 3.4 Construction of nomogram and evaluation of prognostic model

The results of the independent prognostic analysis revealed a total of four significant factors (age, pathologic-M, risk score, and pathologic-N) (Supplementary Figures S2A,B). Subsequently, we integrated these prognostic factors to construct a more user-friendly nomogram. The nomogram, based on the identified prognostic factors, was developed to predict the prognosis of CRC patients (1-, 3-, and 5-year survival probabilities) (Supplementary Figure S2C). The accuracy of the nomogram was validated by the calibration curves, which demonstrated high predictive performance. The calibration curves indicate that the predicted 1-, 3-, and 5-year survival probabilities closely align with the theoretical values, confirming the high prediction accuracy of the clinical prognostic model (Supplementary Figure S2D).

### 3.5 Analysis of functional differences and chemotherapy drug sensitivity between risk subgroups

The functional enrichment analysis of the two risk subgroups showed that the GO entries such as vascular endothelial growth factor binding, elastic fiber, and others were mainly enriched in the high-risk group, while lysophosphatidic acid phosphatase activity, and ubiquinone biosynthesis complex were mainly enriched in the low-risk group (Supplementary Figure S3A, Supplementary Table S4). The results of the KEGG pathway exhibited that the high-risk group was enriched for ECM receptor interaction, and leukocyte transendothelial migration, whereas the low-risk group was enriched for propanoate metabolism and butanoate metabolism (Supplementary Figure S3B, Supplementary Table S5). Functional enrichment analysis revealed that metabolic pathways were significantly enriched in the high-risk subgroup. These findings suggest that metabolic dysregulation may contribute to tumor aggressiveness and poor prognosis in CRC, consistent with the well-documented role of metabolic reprogramming in cancer progression (Dong et al., 2022). For advanced CRC, treatments targeting metabolic pathways may have therapeutic implications.

In the enrichment results, no significantly enriched telomere-related entries were found for these three genes in any KEGG pathway, which is likely due to the limited coverage of telomere-related pathways in the KEGG database; however, in the GO enrichment analysis, entries related to telomere maintenance and telomerase regulation were significantly enriched for all three genes. *HSPA1A* was enriched in 19 telomere-related GO terms, with the core terms including telomerase RNA localization, positive regulation of telomere maintenance, and the telomere elongation pathway (NES <1, adjusted *P* value <0.05, Supplementary Figure S3A). *PDE1B* was enriched in 13 terms, with telomerase RNA localization and the telomerase holoenzyme complex being particularly significant (NES <1, adjusted *P* value <0.05, Supplementary Figure S3B). *TFAP2B* was enriched in 12 terms, with telomerase RNA localization and telomere maintenance associated with the DNA damage response both reaching significant levels (NES <1, adjusted *P* value <0.05, Supplementary Figure S3C). The NES values for the telomere-related GO entries of these three genes were all negative, suggesting that the lower the expression levels of these genes, the higher the enrichment of telomere maintenance and telomerase regulation pathways (Supplementary Table S6). This indicates that *PDE1B, TFAP2B, and HSPA1A* may be involved in maintaining telomere homeostasis in colorectal cancer through negative regulatory mechanisms.

To investigate differences in the sensitivity of chemotherapy drugs between the two risk subgroups, we computed IC_50_ for 367 chemotherapy drugs in both groups. Drug sensitivity analysis revealed that the two different risk groups possessed significantly different sensitivities to 205 drugs (A.770,041_55, ACY.1215_264, AS605240_224, AT7867_356, AZ628_29) (Supplementary Table S7). The nine drugs with the lowest *P* values were Dasatinib_51, WH-4-023_56, TGX221_94, and AZD6482_156 (Supplementary Figure S3C). The sensitivity to 5 Fluorouracil was higher in the low-risk group.

### 3.6 The patients in different risk groups show different immune status

**FIGURE 5:**
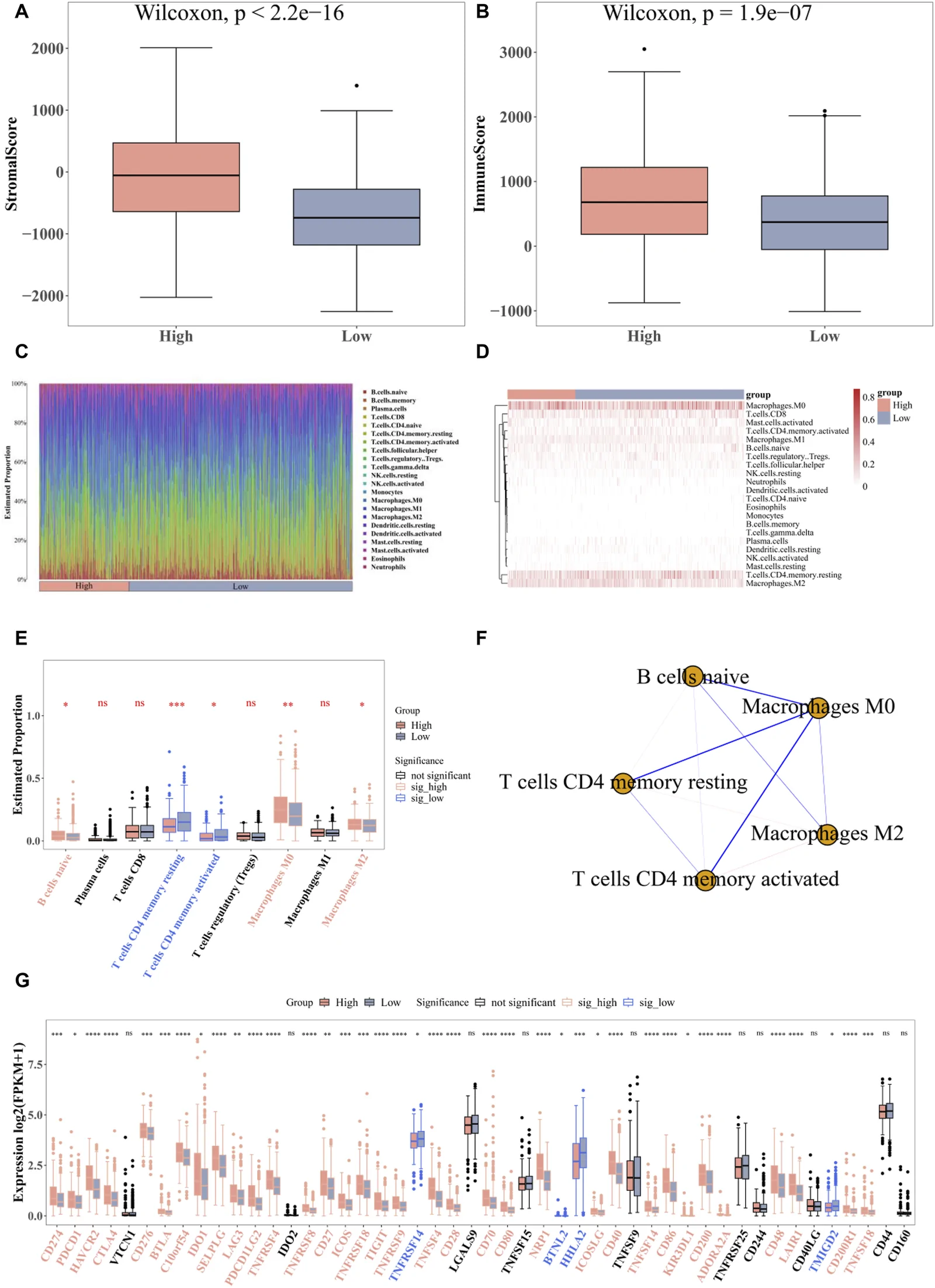
Immune-related analysis between high-and low-risk groups. **(A,B)** Differential analysis of stromal and immune scores. **(C,D)** Histogram of abundance of immune cell infiltration. **(E)** Box plot of immune infiltration differential analysis. **(F)** Correlation network plot of among differential immune cells. **(G)** Box plot of difference in immune checkpoints expression between high-and low-risk groups.

To characterize the distinct tumor immune microenvironment (TIME) landscapes associated with our TMRG-based risk signature, we performed a comprehensive immune cell deconvolution analysis on bulk RNA-seq data. This analysis aims to link the prognostic stratification power of our model to underlying immunological differences between high- and low-risk patient subgroups, thereby providing a broader biological context for its clinical relevance. The results demonstrated that stromal and immune scores were higher in the high-risk group (Figures 5A,B). Figure 5C displays the relative proportions of 22 immune cell types across all samples as stacked bar plots. Figure 5D presents a heatmap showing higher infiltration of M0 macrophages, resting memory CD4^+^ T cells, and M2 macrophages in high-risk samples. Figure 5E illustrates the five immune cell types with significantly different infiltration levels between risk groups through violin plots. Among them, M0 macrophages, naive B cells and M2 macrophages were more infiltrated in the high-risk group, while resting and activated memory CD4 T cells were the opposite. The correlation analysis indicated that there was a significant negative correlation between M0 macrophages with naive B cells, resting and activated memory CD4 T cells (Figure 5F).

The differential analysis revealed that there were 38 differential immune checkpoints (primarily HAVCR2, CD40, PDCD1LG2 and others) between the two risk subgroups (Figure 5G). Among these, four immune checkpoints were downregulated in the high-risk group, while 34 were upregulated in the low-risk group.

In addition, the tumor immune dysfunction and exclusion (TIDE), Dysfunction, Exclusion scores was higher in the high-risk group according to the TIDE algorithm (Supplementary Figure S4A). This suggested that patients in the high-risk group responded less well to immunotherapy than those in the low-risk group. The correlation analysis revealed that Dysfunction had the highest correlation (cor = 0.32, *p* = 4.2e-16) with risk scores (Supplementary Figures S4B–D).

Finally, the Immune Prognostic Score (IPS) analysis illustrated that except for the IPS score, the remaining 3 scores were significantly different between the two risk subgroups and were higher in the low-risk group (Supplementary Figures S4E–H). This suggests that the immune response was better in the low-risk group after the use of immunosuppressants.

### 3.7 Overexpression PDE1B inhibited CRC cell proliferation, metastasis and induced apoptosis

The expression and localization of three prognostic biomarkers (*PDE1B, TFAP2B, and HSPA1A*) were then analyzed. Chromosomal localization analysis demonstrated that these biomarkers were located on autosomes (Supplementary Figure S5A). In the training set, the expression levels of the biomarkers in the CRC group were significantly lower than those in the control group, with statistical significance determined by unpaired t-test (*P* < 0.05) (Supplementary Figures S5B–D). Several studies have shown that *TFAP2B* and *HSPA1A* are involved in CRC progression (Dong et al., 2022; Hegde et al., 2013), which is consistent with our study. The prognostic role of *PDE1B* in CRC, however, appears to be context-dependent. While our analysis of the TCGA-CRC cohort identified low *PDE1B* expression as significantly associated with poor prognosis, this association was not consistently replicated across all publicly available datasets. For instance, an analysis of the Human Protein Atlas (HPA) database did not classify *PDE1B* as a prognostic marker in CRC. To address this discrepancy, we performed a systematic validation across multiple independent GEO datasets (GSE17536, GSE17537, etc). Among these, only the GSE72970 dataset (n = 121) confirmed a significant association between high *PDE1B* expression and better overall survival (log-rank *p* < 0.05; Supplementary Figure S8). Furthermore, our analysis revealed that PDE1B has a relatively low baseline expression level in CRC tissues (baseMean = 69.73). Consistent with its known tissue specificity, GEPIA database analysis showed that PDE1B is predominantly expressed in brain tissues across the human body (Supplementary Figure S9).

Critically, to determine the cellular source of the *PDE1B* signal in the tumor bulk, we analyzed the single-cell RNA sequencing (scRNA-seq) dataset GSE161277. Our analysis revealed that *PDE1B* expression is primarily localized to tumor-infiltrating immune cells, specifically T cells and myeloid cells, with only minimal expression detected in malignant epithelial cells (Supplementary Figure S10). The observed differences likely arise from the inherent nature of bulk RNA-seq, which measures an aggregate signal across heterogeneous cell populations including malignant epithelial cells, stromal components, and tumor-infiltrating immune cells making overall expression levels susceptible to compositional bias, particularly from variable immune infiltration. Collectively, these observations suggest that the prognostic association of *PDE1B* observed in our bulk-level analyses may be largely driven by the composition of the tumor immune microenvironment, rather than reflecting intrinsic expression within the cancer cell compartment itself.

Despite this, the robust correlation between *PDE1B* expression and patient survival in our primary cohort, coupled with its established role as a key regulator of cyclic nucleotide signaling, led us to hypothesize that *PDE1B* may possess direct, cell-autonomous functions in regulating core cancer cell phenotypes. To test this hypothesis and isolate the effect of *PDE1B* from the influence of the tumor microenvironment, we conducted functional validation experiments using established CRC cell lines (HCT116 and SW480), which represent a pure population of malignant epithelial cells.

**FIGURE 6:**
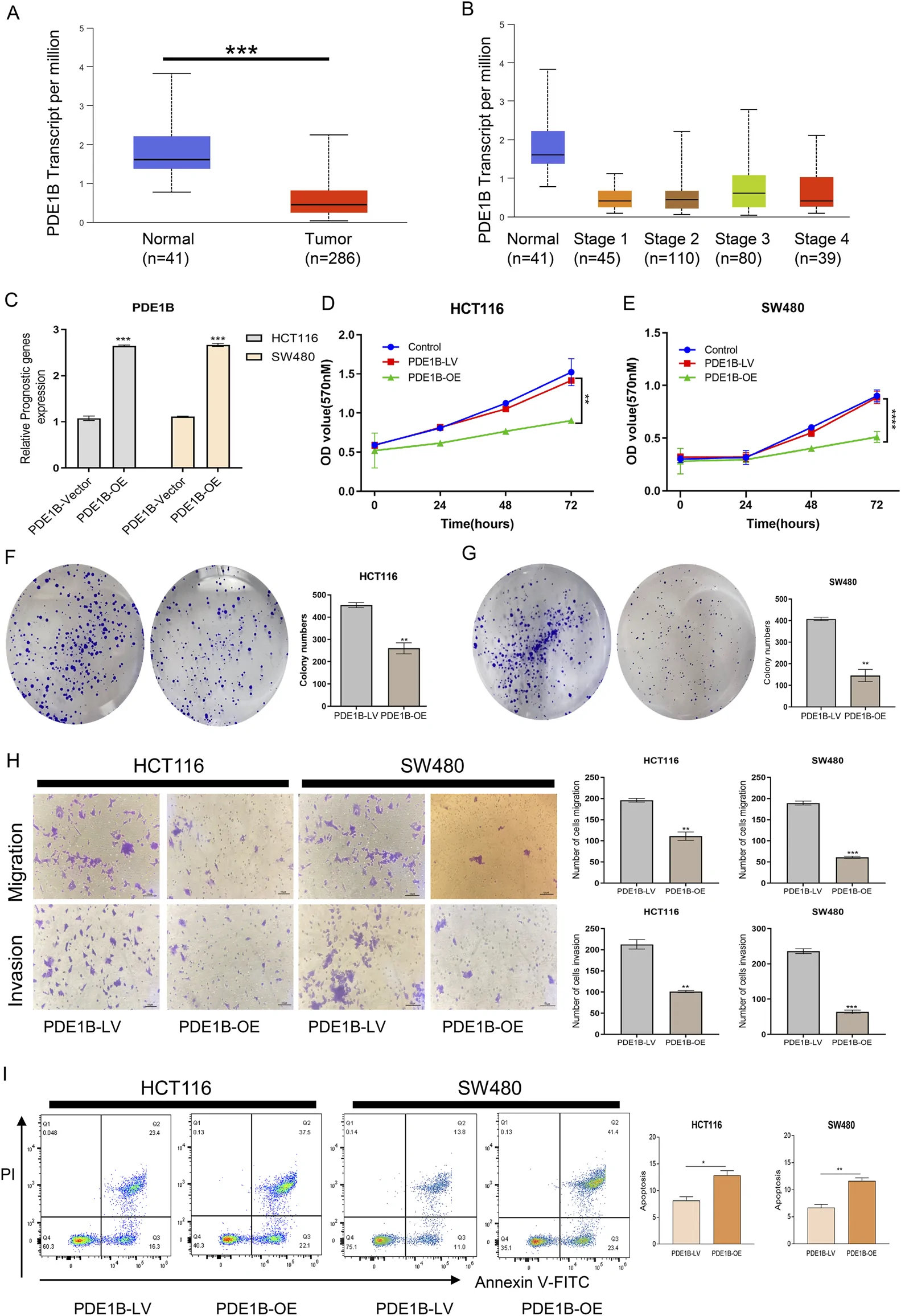
Overexpression of PDE1B inhibits CRC cell proliferation, metastasis and induces apoptosis. **(A)** PDE1B expression significantly lower in CRC tissues (n = 517) compared to normal colon tissues (n = 51) (*P* < 0.001). **(B)** PDE1B expression negatively correlated with pathological grade (r = −0.42, *P* < 0.001). **(C)** qRT-PCR confirmed ∼1.5-fold increase in PDE1B mRNA levels in PDE1B-overexpressing cells (n = 3, *P* < 0.05). **(D,E)** MTT assays showed significantly reduced cell viability in PDE1B-overexpressing cells (HCT116: *P* < 0.01; SW480: *P* < 0.001). **(F,G)** Colony formation assays demonstrated significantly reduced colony numbers in PDE1B-overexpressing cells (HCT116: *P* < 0.001; SW480: *P* < 0.01). **(H)** Transwell assays revealed significantly reduced migration (*P* < 0.001) and invasion (*P* < 0.01) in PDE1B-overexpressing cells. **(I)** Flow cytometry showed significantly increased apoptosis rates in PDE1B-overexpressing cells (*P* < 0.001). Technical details: Data presented as mean ± SD; *P < 0.05, **P < 0.01, ***P < 0.001; all experiments performed in triplicate. Legend: NC, negative control; PDE1B-OE, PDE1B overexpression.

To provide an independent assessment of *PDE1B* expression patterns using a user-friendly platform, we queried the UALCAN database, which utilizes TCGA data. Consistent with our own analysis, UALCAN confirmed that PDE1B mRNA levels are significantly lower in CRC tumor tissues compared to normal tissues (Figure 6A, *P* < 0.001). However, an analysis of PDE1B expression across different pathological stages using the same UALCAN/TCGA data did not reveal a statistically significant trend (Figure 6B). Therefore, while PDE1B is downregulated in CRC overall, its expression level does not appear to be a robust indicator of disease progression stage within the TCGA cohort. Given these limitations of the bulk tissue data, our functional validation in pure CRC cell lines becomes crucial for establishing the direct biological role of PDE1B in carcinogenesis. Following this, we overexpressed PDE1B in HCT116 and SW480 cell lines using lentiviral transduction. qPCR analysis confirmed approximately a 1.5-fold increase in PDE1B mRNA levels relative to negative controls (Figure 6C).

MTT assays revealed that overexpression of PDE1B significantly decreased cell viability in both HCT116 and SW480 cells compared to negative controls (Figures 6D,E). Furthermore, colony formation assays demonstrated that *PDE1B* overexpression markedly reduced the colony-forming ability of these cells (Figures 6F,G). Transwell assays further indicated that upregulated PDE1B expression suppressed both migration and invasion capabilities compared to negative controls (Figure 6H). Flow cytometry results exhibited that the PDE1B-overexpressing group exhibited a significantly higher proportion of apoptotic cells compared to control cells (Figure 6I), suggesting that *PDE1B* overexpression promotes apoptosis in CRC cells. Collectively, these findings indicate that PDE1B is involved in CRC carcinogenesis and may serve as a prognostic biomarker and a potential therapeutic target.

Collectively, these findings reveal a dual aspect of PDE1B in CRC: its bulk tissue expression serves as a prognostic indicator potentially linked to the immune contexture, while the protein itself functions as a potent tumor-suppressive regulator when expressed within cancer cells. This underscores the importance of integrating bulk transcriptomic analysis with single-cell resolution and functional validation to fully understand gene function in complex diseases like cancer.

## 4 Discussion

Colorectal cancer continues to be one of the leading malignancies and causes of cancer-related deaths worldwide. It is widely recognized that the prognosis of CRC patients is influenced by tumor infiltration in distant organs and lymph node metastasis. Telomeres have a primary function of maintaining genomic stability, which plays a critical role in cellular immortalization, a process that contributes significantly to tumorigenesis, infiltration and metastasis (Hegde et al., 2013, Min and Shay, 2016, Tatsumoto et al., 2000). Hence, telomeres serve a crucial function in the progression of CRC (Stewénius et al., 2005, Tahara et al., 1995, Terrin et al., 2008; Ren et al., 2022, Xiao et al., 2023). This study provides a CRC-specific analysis of telomere maintenance-related genes, validating their prognostic significance in the context of colorectal cancer.

In this study, 976 differentially expressed TMRGs were identified enriched in telomere maintenance and DNA replication pathways. Through Cox regression algorithms, we developed and validated a risk model comprising three telomere maintenance-related biomarkers (*PDE1B, TFAP2B, and HSPA1A*). This model effectively stratifies patients into distinct risk subgroups with divergent survival outcomes, immune microenvironment characteristics, and chemotherapeutic sensitivities. Furthermore, our nomogram integrating risk scores with clinical parameters (pathologic N/M stage, age) demonstrated high accuracy in predicting 1-, 3-, and 5-year survival rates. Immune profiling revealed differential infiltration of M0/M2 macrophages and CD4^+^ T cells between risk subgroups, suggesting distinct immune evasion mechanisms. Drug sensitivity analysis highlighted enhanced 5-fluorouracil efficacy in low-risk patients, providing actionable insights for personalized therapy.

The three signature genes have been implicated in distinct biological processes linked to telomere homeostasis and tumor progression. *HSPA1A*, a member of the HSP70 family, functions as a molecular chaperone that facilitates proper protein folding and maintains proteostasis under cellular stress (Lackie et al., 2017; Gupta et al., 2020). Beyond its canonical role, emerging evidence suggests HSPA1A contributes to telomere maintenance through direct interaction with shelter in complex components and protection of telomeric DNA from oxidative damage (Dong et al., 2025; Daniels et al., 2022). Our finding that *HSPA1A* is downregulated in CRC aligns with reports of its tumor-suppressive function in gastrointestinal cancers (Guan et al., 2021). Critically, *HSPA1A* knockout models exhibit increased chromosomal aberrations and genomic stability (Hunt et al., 2004), phenotypes directly linked to telomere dysfunction. This positions HSPA1A as a guardian of telomere integrity under genotoxic stress. The study reported that *HSPA1A* was downregulated in CRC patients, and increased expression of *HSPA1A1* was associated with a poor prognosis, which was consistent with our conclusion. Furthermore, it was found by Hunt et al. that knocking out *HSPA1A* in mouse fibroblasts resulted in increased levels of spontaneous and radiation-induced chromosomal aberrations. This suggests that the *HSPA1A* protein may play a role in maintaining genome stability (Hunt et al., 2004). *HSPA1A* is associated with cancer cell proliferation and promotes the proliferation of H22 hepatocellular carcinoma cells through TLR2 and TLR4 signaling. It is hypothesized that *HSPA1A* acts as an endogenous ligand for TLR2 and TLR4 to promote tumor growth (Wu et al., 2012).

*TFAP2B*, a developmentally regulated transcription factor, has been implicated in epithelial-mesenchymal transition (EMT) and metastatic progression across multiple carcinomas (Wu and Zhang, 2018; Raap et al., 2018). While its role in CRC remains underexplored, recent studies demonstrate that TFAP2 family members can transcriptionally regulate telomerase reverse transcriptase (TERT) expression and modulate DNA damage response pathways (Lu et al., 2016; Fu et al., 2013). In our cohort, *TFAP2B* showed a positive beta coefficient in the Cox regression model (coef = 0.9323, HR = 2.5404, *p* = 0.0015), while low *TFAP2B* expression still correlates with advanced pathological stages, suggesting its potential involvement in restraining aggressive tumor phenotypes may be mediated by indirect regulatory mechanisms (e.g., tumor microenvironment remodeling) rather than a direct prognostic effect, which warrants further mechanistic exploration.

Notably, among the three identified biomarkers, we prioritized PDE1B for functional validation based on two key considerations. First, PDE1B, a member of the phosphodiesterase (PDE) family (Delyon et al., 2017, Lugnier, 2006, Goldhoff et al., 2008) and the PDE1 subfamily, has been increasingly implicated in multiple malignancies (Beavo, 1995). Recent multi-omics and Mendelian randomization analyses identified *PDE1B* as a favorable prognostic factor in CRC (He et al., 2025), while other studies reported its association with the tumor microenvironment and improved clinical outcomes in osteosarcoma (Tan et al., 2021; Chen et al., 2024). Despite this emerging evidence, the specific biological role of *PDE1B* in CRC pathogenesis remains poorly characterized. Second, and more importantly, our prognostic model assigned *PDE1B* a positive regression coefficient (coef = 0.70, HR = 2.01, *p* = 0.008) in multivariate Cox regression analysis, which remained an independent prognostic factor after adjusting for clinical variables. In the context of our risk score formula, this positive coefficient indicates that lower *PDE1B* expression leads to higher risk scores and poorer patient survival outcomes. This result is consistent with accumulating data suggesting a potential tumor-suppressive function of *PDE1B* in diverse cancers (He et al., 2025; Tan et al., 2021; Chen et al., 2024; Zhao et al., 2021). Given the urgent need to identify novel tumor suppressors in CRC, we hypothesized that *PDE1B* may act as a previously unrecognized tumor-suppressive regulator in this disease context. To test this hypothesis, a number of *in vitro* experiments were carried out to investigate its effects in CRC cells. Our results demonstrate that *PDE1B* is expressed at low levels in CRC, with its low expression associated with poor overall survival. Overexpression of *PDE1B* inhibits cell proliferation and metastasis, while promoting apoptosis in CRC cell lines.

The inclusion of these three genes, *PDE1B* (direct telomere regulator), *TFAP2B* (transcriptional modulator of stress response), and *HSPA1A* (telomere protector), in a single prognostic model therefore captures both direct (telomere-specific) and indirect (stress response, genomic stability) dimensions of CRC aggressiveness, which may partially account for its relatively stable predictive performance across validation cohorts. Our additional analyses provide further insight into their relationship. While *HSPA1A* and *TFAP2B* exhibit a significant positive expression correlation and are linked within a shared functional network related to transcriptional control and cellular stress, *PDE1B* appears to act more independently. This lack of strong co-expression or direct physical interaction among all three components suggests that their combined prognostic value stems from their ability to capture distinct, non-redundant aspects of tumor biology, thereby offering a more comprehensive assessment of patient risk than any single gene could provide.

To examine whether our telomere-related signature correlates with established CRC molecular markers (MSI/MMR and APC, KRAS, NRAS, BRAF, TP53), we calculated Spearman correlations between the risk score and 11 marker genes. Using |cor| > 0.3 and *p* < 0.05 as the significance threshold for meaningful effect size, none of the markers qualified. While APC (0.16), BRAF (0.15), and TP53 (−0.15) were statistically significant, all coefficients remained below 0.3, reflecting only weak associations. MSH2 (−0.11) and MLH3 (0.10) also did not reach the threshold, and the rest showed no significance. Collectively, these results suggest that our risk score is independent of classic molecular subtypes, implying that it may capture prognostic information beyond existing classifications. This independence is biologically plausible for several reasons: (i) driver mutations and telomere genes act through distinct pathways (initiation vs. progression); (ii) our score predicts survival independently of conventional markers; and (iii) its independence from existing subtypes adds complementary prognostic value, without undermining our core conclusions.

Our immune profiling revealed a paradoxical yet clinically coherent pattern in the high-risk subgroup: despite elevated immune cell infiltration (particularly M0/M2 macrophages), these tumors exhibited impaired anti-tumor immunity characterized by reduced CD4^+^ memory T cells and upregulated immune checkpoint molecules. Specifically, 34 of 48 immune checkpoints, including HAVCR2 (TIM-3), PDCD1LG2 (PD-L2), and CD40, were significantly overexpressed in high-risk tumors, indicative of an adaptive immune resistance mechanism whereby tumors co-opt checkpoint pathways to suppress T-cell function (Li et al., 2024; Cao et al., 2024).

This molecular signature of immune exhaustion directly aligns with our computational prediction of poor immunotherapy response, as evidenced by significantly higher TIDE, Dysfunction, and Exclusion scores in the high-risk group. The TIDE algorithm quantifies two key barriers to immunotherapy efficacy: T-cell dysfunction (impaired cytotoxic activity despite infiltration) and T-cell exclusion (failure of T-cells to penetrate the tumor core) (Zhan et al., 2025). The strong positive correlation between risk scores and TIDE Dysfunction scores (r = 0.32, *p* = 4.2e-16) provides a mechanistic bridge linking checkpoint overexpression to functional T-cell impairment.

Collectively, these findings elucidate a context-dependent duality in the function of *PDE1B* in CRC, effectively reconciling the expression discrepancies observed between our bulk-tissue and single-cell multi-omics analyses. In bulk tumor specimens, *PDE1B* transcript abundance is primarily derived from infiltrating T cells and myeloid immune cells, rather than from the malignant epithelial compartment. Conversely, cell-intrinsic investigations using purified epithelial cell lines demonstrate that forced *PDE1B* overexpression directly curbs proliferation and metastasis while promoting apoptosis in colorectal cancer cells, confirming its cell-autonomous tumor-suppressive activity that is uncoupled from immune-derived signals. This dichotomous functionality provides a compelling explanation for why *PDE1B*, despite exhibiting extremely low endogenous expression in tumor epithelia, retains potent prognostic stratification power in bulk-tissue cohorts. Indeed, its bulk-level expression signature serves as a dual-purpose readout, concurrently capturing the state of the TIME and the activity of intrinsic tumor-suppressive pathways within the neoplastic epithelium.

Most published prognostic models for colorectal cancer have relied on a relatively large set of core genes to construct risk scores, for example, the immunotherapy resistance-related model (Ma et al., 2025) and the six-gene glutamine signature model (Yin et al., 2026). By contrast, our model incorporates only three independent prognostic genes, *PDE1B, TFAP2B, and HSPA1A*, which substantially reduces assay costs and facilitates clinical translation. Furthermore, the vast majority of existing non-telomere-based prognostic models have been confined to transcriptome-level screening without dedicated *in vitro* functional validation of the core genes. For instance, one tumor immune cell signature was derived solely from bulk RNA-seq differential analysis, without any cellular phenotypic verification (Zuo et al., 2025). A few chemotherapy-response models have performed overexpression or knockdown experiments for only a single core gene (Zhou et al., 2025), and very few prognostic models have conducted a complete panel of functional assays, covering proliferation, migration, invasion, and apoptosis—for a single gene, as we have done here for *PDE1B*. In addition, we constructed a comprehensive nomogram that integrates the risk score, N/M stage, and patient age. However, similar to nearly all retrospective omics-based prognostic models, this nomogram shares the common limitation of lacking prospective multicenter clinical validation.

We calculated the IC_50_ of 367 chemotherapeutic drugs and analyzed the differences in IC_50_ values between the high- and low-risk groups, which showed that the high-risk group was more sensitive to 9 drugs, including pazopanib, but the low-risk group was more sensitive to 5 fluorouracil and CAY10566_416, which suggests that we should use the corresponding drugs for different conditions in order to obtain better therapeutic effects.

The prognostic clinical model developed in this study, combined with subtype analysis in colorectal cancer, demonstrates potential for improving tumor progression prediction, guiding clinical decision-making, and facilitating personalized medicine and immunotherapy strategies. While the predictive performance of the nomograms has been thoroughly validated, further practical clinical studies are required to establish a robust theoretical foundation for real-world application. Specifically, the risk stratification provided by this model can assist clinicians in three key areas: (1) determining appropriate follow-up intervals (high-risk patients may require more frequent monitoring, e.g., every 3–6 months, compared to low-risk patients who may be monitored annually); (2) selecting optimal adjuvant chemotherapy regimens (low-risk patients showed higher sensitivity to 5-fluorouracil, suggesting they may benefit from standard regimens, while high-risk patients may require more aggressive treatment strategies such as FOLFOX or FOLFIRI); and (3) identifying patients who may benefit from immunotherapy (low-risk patients demonstrated better immune microenvironment characteristics, including higher immune scores and lower TIDE scores, suggesting they may respond better to immune checkpoint inhibitors).

Several limitations of this study warrant consideration. First, functional validation was performed exclusively for *PDE1B*; the tumor-related phenotypes of *TFAP2B* and *HSPA1A* were primarily corroborated by our transcriptomic data and previously published cellular and animal studies, rather than by dedicated experiments within our own experimental system. Second, the *PDE1B*-based prognostic signature derived from bulk RNA-seq is subject to cell-type composition bias. RNA-seq data revealed that *PDE1B* is predominantly expressed in tumor-infiltrating immune cells, whereas bulk sequencing cannot discriminate between transcripts originating from immune cells versus those from malignant epithelial cells, thereby hindering precise mechanistic interpretation of its prognostic role. Future studies employing laser capture microdissection or primary epithelial cell sorting from colorectal cancer tissues will be required to purify the epithelial compartment, assess *PDE1B* expression without immune-cell contamination, and validate its independent prognostic value. Third, both the risk model and the integrated nomogram were constructed and internally validated solely using publicly available retrospective transcriptomic cohorts, without prospective or multicenter clinical datasets. Therefore, the present findings do not yet provide sufficient evidence to support that our model can optimize or supplant current guideline-based therapeutic strategies; the conclusions drawn are exploratory and not immediately generalizable to clinical practice. Prospective clinical trials with concurrent transcriptomic profiling and long-term follow-up are warranted to further evaluate the clinical utility of this model in patient risk stratification and individualized treatment guidance.

## 5 Conclusion

In this study, we constructed a robust telomere maintenance-related gene signature (*PDE1B*, *TFAP2B*, and *HSPA1A*) as an independent prognostic predictor in CRC. The risk stratification model effectively discriminates patients with distinct survival patterns, tumor microenvironments, and therapeutic responses, providing a valuable tool for clinical decision-making. Specifically, this model provides complementary molecular risk information and is not intended to replace established clinical parameters currently used in routine colorectal cancer management. As an exploratory bioinformatics tool developed on the basis of retrospective public cohorts, it is not yet capable of independently altering standardized postoperative surveillance or adjuvant treatment protocols at the present stage. Our findings have shown that the *PDE1B* gene was expressed at low levels in CRC, and its low expression was linked to poor overall survival, and overexpression *PDE1B* inhibited cell proliferation and metastasis, while promoting apoptosis in CRC cell lines. These results offer valuable new insights for future research seeking to utilize telomere maintenance-related genes in the fight against CRC and to assess prognosis.

## Data Availability

The datasets generated and/or analyzed during the current study are available in the GEO and TCGA repository. The GSE28722 and GSE28722 were obtained from the GEO database (https://www.ncbi.nlm.nih.gov/geo/), and the TCGA-CRC were downloaded from the TCGA database (https://portal.gdc.cancer.gov/).

## Supplementary material

SUPPLEMENTARY FIGURE S1 Clinicopathological features and survival analysis between two risk subtypes.

SUPPLEMENTARY FIGURE S2 Construction and evaluation of nomogram. **(A)** Univariate independent prognostic analysis forest plot. **(B)** Multifactorial independent prognostic forest plot. **(C)** 1, 3, 5 years calibration curve. **(D)** Nomogram.

SUPPLEMENTARY FIGURE S3 Gene set enrichment analysis of telomere-related GO terms for prognostic genes.

SUPPLEMENTARY FIGURE S4 Pathway enrichment of KEGG and GO and chemotherapy drug sensitivity analysis in two risk subgroups. **(A)** Heat map of the GO entries difference. **(B)** Heat map of the KEGG pathway difference. **(C)** Boxplot of Chemotherapy drug sensitivity analysis.

SUPPLEMENTARY FIGURE S5 Immune-related analysis between high-and low-risk groups. **(A)** Box plot of difference analysis in TIDE, Dysfunction, and Exclusion scores. **(B–D)**. Scatterplot of correlation analysis. **(E–H)**. Box plot of IPS score difference analysis.

SUPPLEMENTARY FIGURE S6 Expression analysis of prognostic biomarkers. **(A)** Chromosomal locations of the three biomarkers on autosomes. **(B–D)** Expression levels of PDE1B, TFAP2B, and HSPA1A in CRC (n = 638) versus control (n = 51) groups, showing significant downregulation in CRC (**(B)** P = 0.002; **(C)** P = 0.008; **(D)** P = 0.004). Technical details: Data presented as mean ± SD; **P* < 0.05, ***P* < 0.01, ****P* < 0.001. Legend: CRC = colorectal cancer; Control = normal adjacent tissues.

SUPPLEMENTARY FIGURE S7 Assessment of batch effects in the TCGA-CRC cohort using Principal Component Analysis (PCA).

SUPPLEMENTARY FIGURE S8 Performance evaluation of the prognostic risk model.

SUPPLEMENTARY FIGURE S9 Prognostic validation of PDE1B in an independent CRC cohort (GSE72970).

SUPPLEMENTARY FIGURE S10 Pan-cancer and pan-tissue expression profile of PDE1B.

SUPPLEMENTARY FIGURE S11 Single-cell RNA sequencing analysis reveals the cellular origin of PDE1B expression in colorectal cancer.

SUPPLEMENTARY FIGURE S12 Pairwise correlation analysis of the three signature genes.

SUPPLEMENTARY FIGURE S13 Protein-protein interaction (PPI) network of the three signature genes.
