# Correction: Crucial role of telomere maintenance-related genes in survival prediction and subtype identification in colorectal cancer

**DOI:** 10.3389/fmolb.2026.2013379

**Published:** 2026-10-26

**Authors:** Feng Huang, Jung-Yin Fong, Chin Tat Ng, Karthikkumar Venkatachalam, Mohammed Abdullah Alshawsh, Chun-Wai Mai, Yik-Ling Chew, Malarvili Selvaraja

**Affiliations:** 1 Faculty of Pharmaceutical Sciences, UCSI University, Kuala Lumpur, Malaysia; 2 Faculty of Applied Sciences, UCSI University, Cheras, Malaysia; 3 Faculty of Medicine, Universiti Kebangsaan Malaysia, Kuala Lumpur, Malaysia; 4 Centre for Cancer Prevention and Drug Development, Department of Internal Medicine, Hematology-Oncology Section, The University of Oklahoma Health Sciences, Oklahoma City, OK, United States; 5 Faculty of Medicine, Nursing and Health Sciences, Monash University, Clayton, VIC, Australia; 6 Department of Pharmacology, Universiti Malaya Faculty of Medicine, Kuala Lumpur, Malaysia; 7 Centre for Cancer and Stem Cell Research, Institute of Research, Development and Innovation (IRDI), IMU University, Kuala Lumpur, Malaysia; 8 UCSI University Faculty of Pharmaceutical Sciences, Cheras, Malaysia

**Keywords:** colorectal cancer, drug sensitivity, immune infiltration, PDE1B, prognostic model, telomere maintenance-related genes

Author Muralidharan Anbalagan and Ravishankar Ram Mani was erroneously included as an author in the published article.

The correct author list reads:

Feng Huang^1,^*^,†^, Jung-Yin Fong^2^, Chin Tat Ng^3^, Karthikkumar Venkatachalam^4^, Mohammed Abdullah Alshawsh^5,6^, Chun-Wai Mai^7^, Yik-Ling Chew^1^ and Malarvili Selvaraja^1,8,^*^†^

† These authors contributed equally to this work and share first authorship

The Author Contributions Statement has been corrected to read:

FH: Conceptualization, Data curation, Formal Analysis, Investigation, Methodology, Software, Validation, Writing–original draft, Writing–review and editing. J-YF: Writing–original draft. CT: Writing–review and editing. KV: Writing–review and editing, Supervision. MA: Software, Writing–review and editing, Supervision. C-WM: Writing–review and editing. Y-LC: Writing–review and editing. MS: Conceptualization, Data curation, Formal Analysis, Methodology, Project administration, Validation, Supervision, Writing–review and editing.

The original version of this article has been updated.

