# Supplementary material for: Crucial role of telomere maintenance-related genes in survival prediction and subtype identification in colorectal cancer
Source: Front Mol Biosci. 2026 Aug 28;13:1728178. doi: 10.3389/fmolb.2026.1728178 (PMC13561823; doi:10.3389/fmolb.2026.1728178)
Supplement: Supplementary file 1 [file DataSheet1.pdf]

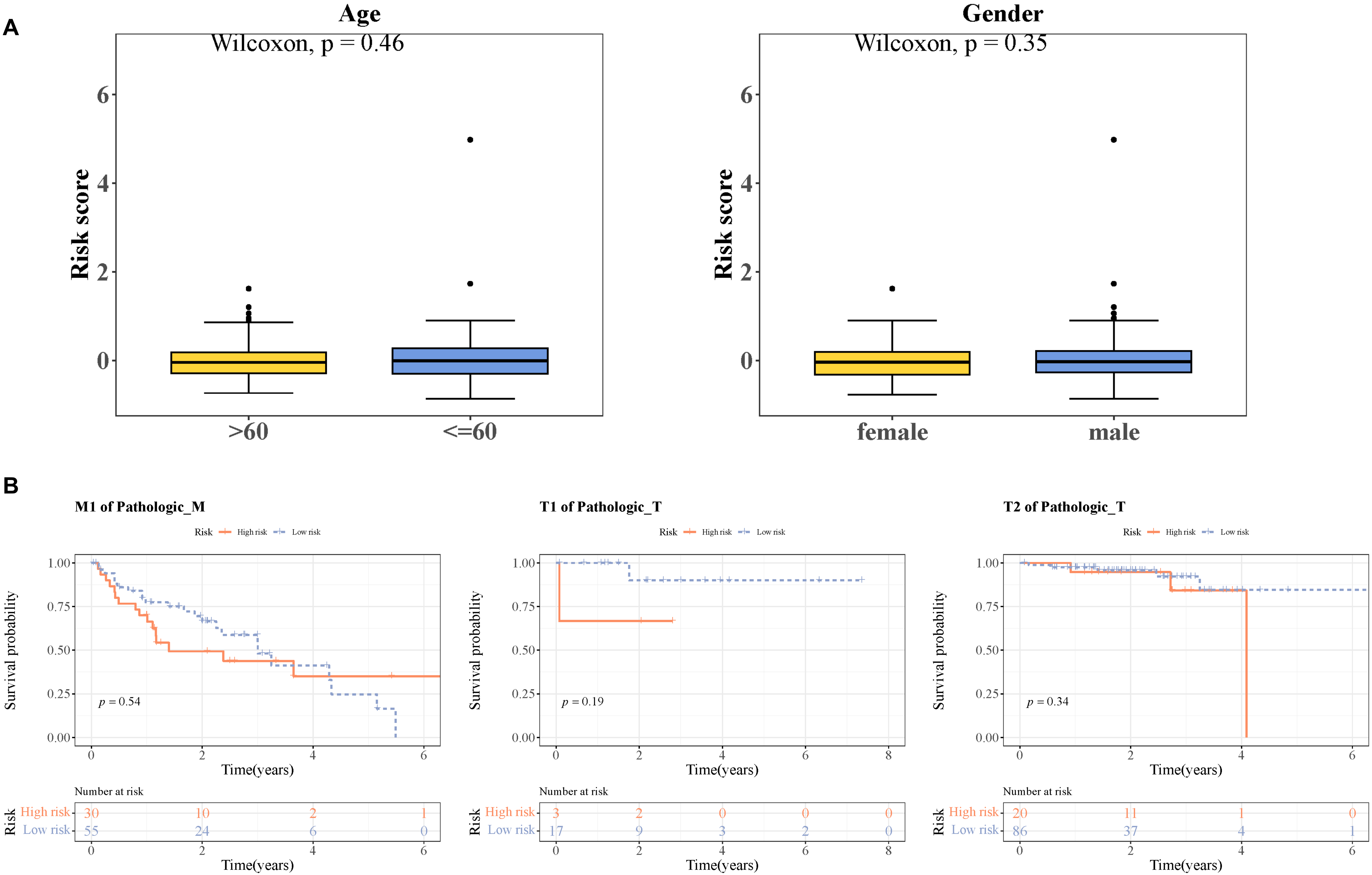

Supplementary Figure 1. Clinical characterization and survival analysis between two risk subgroups. **A.** Boxplot of differential analysis. **B.** KM survival curve between two risk subgroups in patients.
