# Supplementary material for: Crucial role of telomere maintenance-related genes in survival prediction and subtype identification in colorectal cancer
Source: Front Mol Biosci. 2026 Aug 28;13:1728178. doi: 10.3389/fmolb.2026.1728178 (PMC13561823; doi:10.3389/fmolb.2026.1728178)
Supplement: Supplementary file 2 [file DataSheet10.pdf]

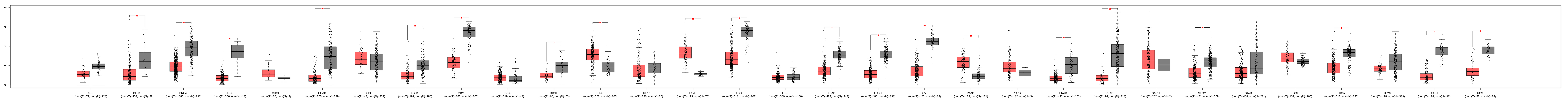

0
2
4
6
8
ACC (num(T)=77; num(N)=128)
BLCA (num(T)=404; num(N)=28)
BRCA (num(T)=1085; num(N)=291)
CESC (num(T)=306; num(N)=13)
CHOL (num(T)=36; num(N)=9)
COAD (num(T)=275; num(N)=349)
DLBC (num(T)=47; num(N)=337)
ESCA (num(T)=182; num(N)=286)
GBM (num(T)=163; num(N)=207)
HNSC (num(T)=519; num(N)=44)
KICH (num(T)=66; num(N)=53)
KIRC (num(T)=523; num(N)=100)
KIRP (num(T)=286; num(N)=60)
LAML (num(T)=173; num(N)=70)
LGG (num(T)=518; num(N)=207)
LIHC (num(T)=369; num(N)=160)
LUAD (num(T)=483; num(N)=347)
LUSC (num(T)=486; num(N)=338)
OV (num(T)=426; num(N)=88)
PAAD (num(T)=179; num(N)=171)
PCPG (num(T)=182; num(N)=3)
PRAD (num(T)=492; num(N)=152)
READ (num(T)=92; num(N)=318)
SARC (num(T)=262; num(N)=2)
SKCM (num(T)=461; num(N)=558)
STAD (num(T)=408; num(N)=211)
TGCT (num(T)=137; num(N)=165)
THCA (num(T)=512; num(N)=337)
THYM (num(T)=118; num(N)=339)
UCEC (num(T)=174; num(N)=91)
UCS (num(T)=57; num(N)=78)
