# Supplementary material for: Crucial role of telomere maintenance-related genes in survival prediction and subtype identification in colorectal cancer
Source: Front Mol Biosci. 2026 Aug 28;13:1728178. doi: 10.3389/fmolb.2026.1728178 (PMC13561823; doi:10.3389/fmolb.2026.1728178)
Supplement: Supplementary file 3 [file DataSheet11.pdf]

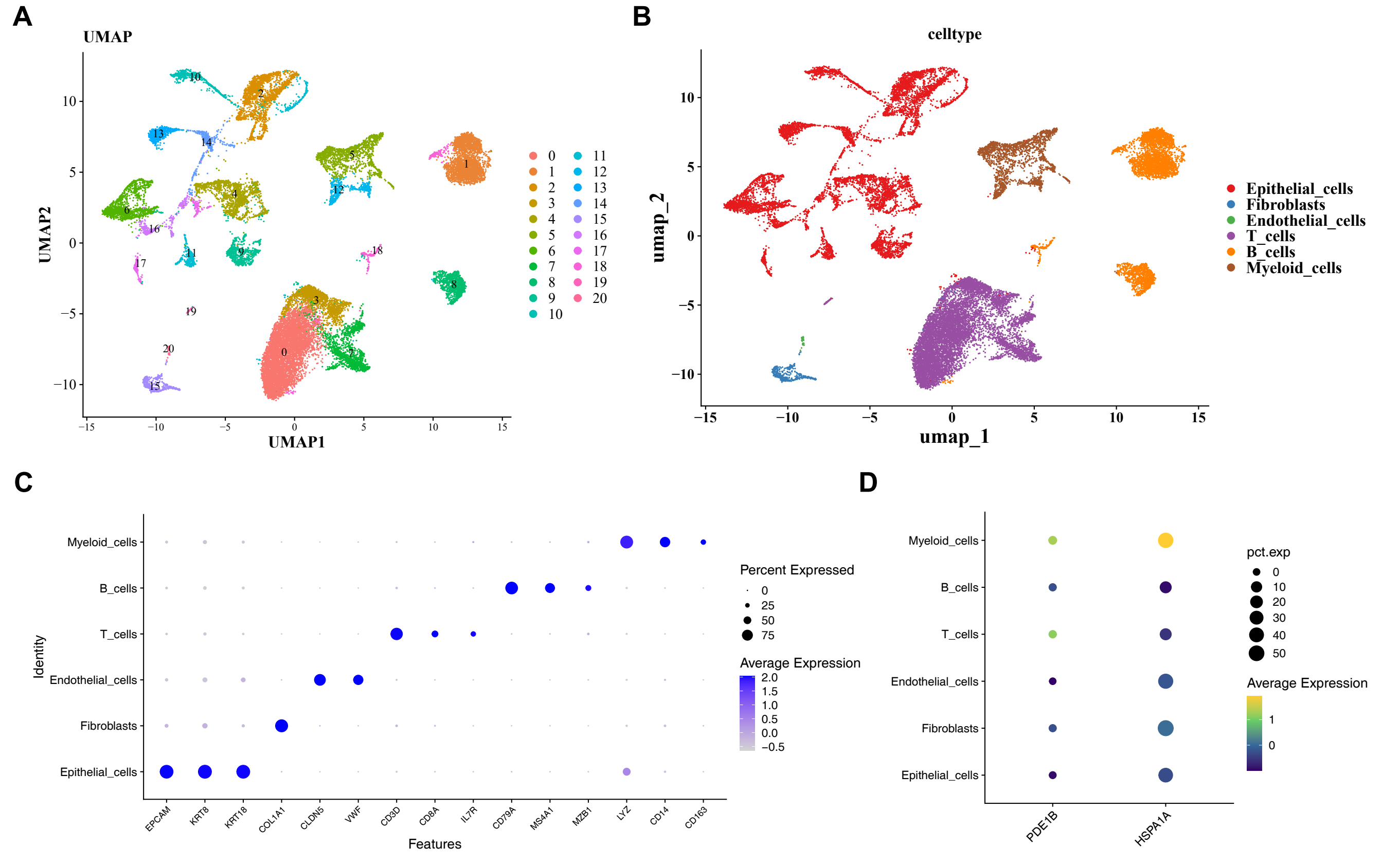
A
UMAP
UMAP1
UMAP2
B
celltype
umap_1
umap_2
Epithelial_cells
Fibroblasts
Endothelial_cells
T_cells
B_cells
Myeloid_cells
C
Identity
Features
Myeloid_cells
B_cells
T_cells
Endothelial_cells
Fibroblasts
Epithelial_cells
EPCAM
KRT8
KRT18
COL1A1
CLDN5
VWF
CD3D
CD8A
IL7R
CD79A
MS4A1
MZB1
LYZ
CD14
CD163
Percent Expressed
Average Expression
D
PDE1B
HSPA1A
pct.exp
Average Expression
