# Supplementary material for: Crucial role of telomere maintenance-related genes in survival prediction and subtype identification in colorectal cancer
Source: Front Mol Biosci. 2026 Aug 28;13:1728178. doi: 10.3389/fmolb.2026.1728178 (PMC13561823; doi:10.3389/fmolb.2026.1728178)
Supplement: Supplementary file 6 [file DataSheet2.pdf]

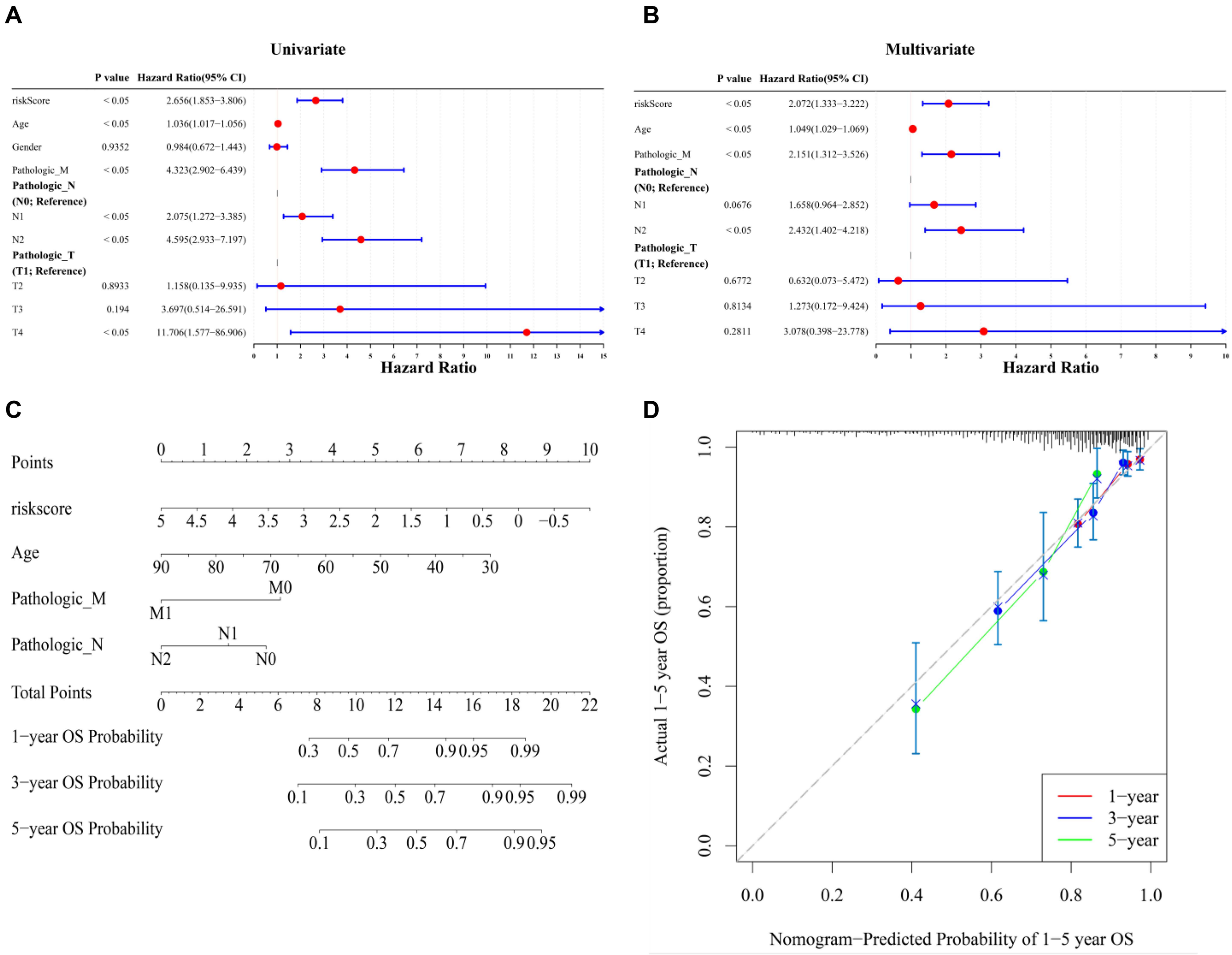
A
Univariate
P value Hazard Ratio(95% CI)
riskScore < 0.05 2.656(1.853−3.806)
Age < 0.05 1.036(1.017−1.056)
Gender 0.9352 0.984(0.672−1.443)
Pathologic_M < 0.05 4.323(2.902−6.439)
Pathologic_N (N0; Reference)
N1 < 0.05 2.075(1.272−3.385)
N2 < 0.05 4.595(2.933−7.197)
Pathologic_T (T1; Reference)
T2 0.8933 1.158(0.135−9.935)
T3 0.194 3.697(0.514−26.591)
T4 < 0.05 11.706(1.577−86.906)
Hazard Ratio
B
Multivariate
P value Hazard Ratio(95% CI)
riskScore < 0.05 2.072(1.333−3.222)
Age < 0.05 1.049(1.029−1.069)
Pathologic_M < 0.05 2.151(1.312−3.526)
Pathologic_N (N0; Reference)
N1 0.0676 1.658(0.964−2.852)
N2 < 0.05 2.432(1.402−4.218)
Pathologic_T (T1; Reference)
T2 0.6772 0.632(0.073−5.472)
T3 0.8134 1.273(0.172−9.424)
T4 0.2811 3.078(0.398−23.778)
Hazard Ratio
C
Points
riskscore
Age
Pathologic_M
M0
M1
Pathologic_N
N1
N2
N0
Total Points
1−year OS Probability
3−year OS Probability
5−year OS Probability
D
Actual 1−5 year OS (proportion)
Nomogram−Predicted Probability of 1−5 year OS
1−year
3−year
5−year
