# Supplementary material for: Crucial role of telomere maintenance-related genes in survival prediction and subtype identification in colorectal cancer
Source: Front Mol Biosci. 2026 Aug 28;13:1728178. doi: 10.3389/fmolb.2026.1728178 (PMC13561823; doi:10.3389/fmolb.2026.1728178)
Supplement: Supplementary file 7 [file DataSheet3.pdf]

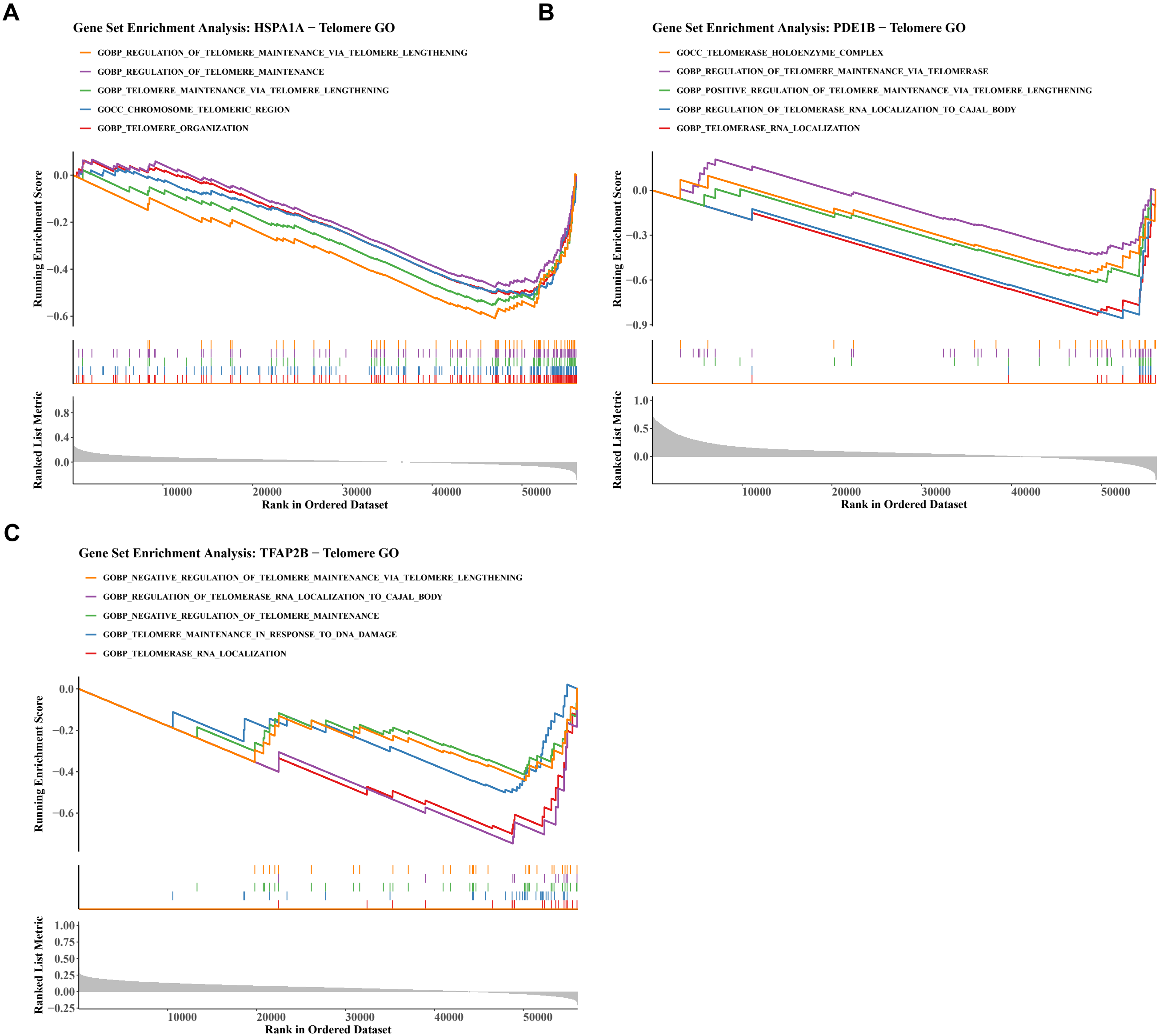

A
Gene Set Enrichment Analysis: HSPA1A − Telomere GO
GOBP_REGULATION_OF_TELOMERE_MAINTENANCE_VIA_TELOMERE_LENGTHENING
GOBP_REGULATION_OF_TELOMERE_MAINTENANCE
GOBP_TELOMERE_MAINTENANCE_VIA_TELOMERE_LENGTHENING
GOCC_CHROMOSOME_TELOMERIC_REGION
GOBP_TELOMERE_ORGANIZATION
Running Enrichment Score
Ranked List Metric
Rank in Ordered Dataset
B
Gene Set Enrichment Analysis: PDE1B − Telomere GO
GOCC_TELOMERASE_HOLOENZYME_COMPLEX
GOBP_REGULATION_OF_TELOMERE_MAINTENANCE_VIA_TELOMERASE
GOBP_POSITIVE_REGULATION_OF_TELOMERE_MAINTENANCE_VIA_TELOMERE_LENGTHENING
GOBP_REGULATION_OF_TELOMERASE_RNA_LOCALIZATION_TO_CAJAL_BODY
GOBP_TELOMERASE_RNA_LOCALIZATION
Running Enrichment Score
Ranked List Metric
Rank in Ordered Dataset
C
Gene Set Enrichment Analysis: TFAP2B − Telomere GO
GOBP_NEGATIVE_REGULATION_OF_TELOMERE_MAINTENANCE_VIA_TELOMERE_LENGTHENING
GOBP_REGULATION_OF_TELOMERASE_RNA_LOCALIZATION_TO_CAJAL_BODY
GOBP_NEGATIVE_REGULATION_OF_TELOMERE_MAINTENANCE
GOBP_TELOMERE_MAINTENANCE_IN_RESPONSE_TO_DNA_DAMAGE
GOBP_TELOMERASE_RNA_LOCALIZATION
Running Enrichment Score
Ranked List Metric
Rank in Ordered Dataset
