# Supplementary material for: Crucial role of telomere maintenance-related genes in survival prediction and subtype identification in colorectal cancer
Source: Front Mol Biosci. 2026 Aug 28;13:1728178. doi: 10.3389/fmolb.2026.1728178 (PMC13561823; doi:10.3389/fmolb.2026.1728178)
Supplement: Supplementary file 8 [file DataSheet4.pdf]

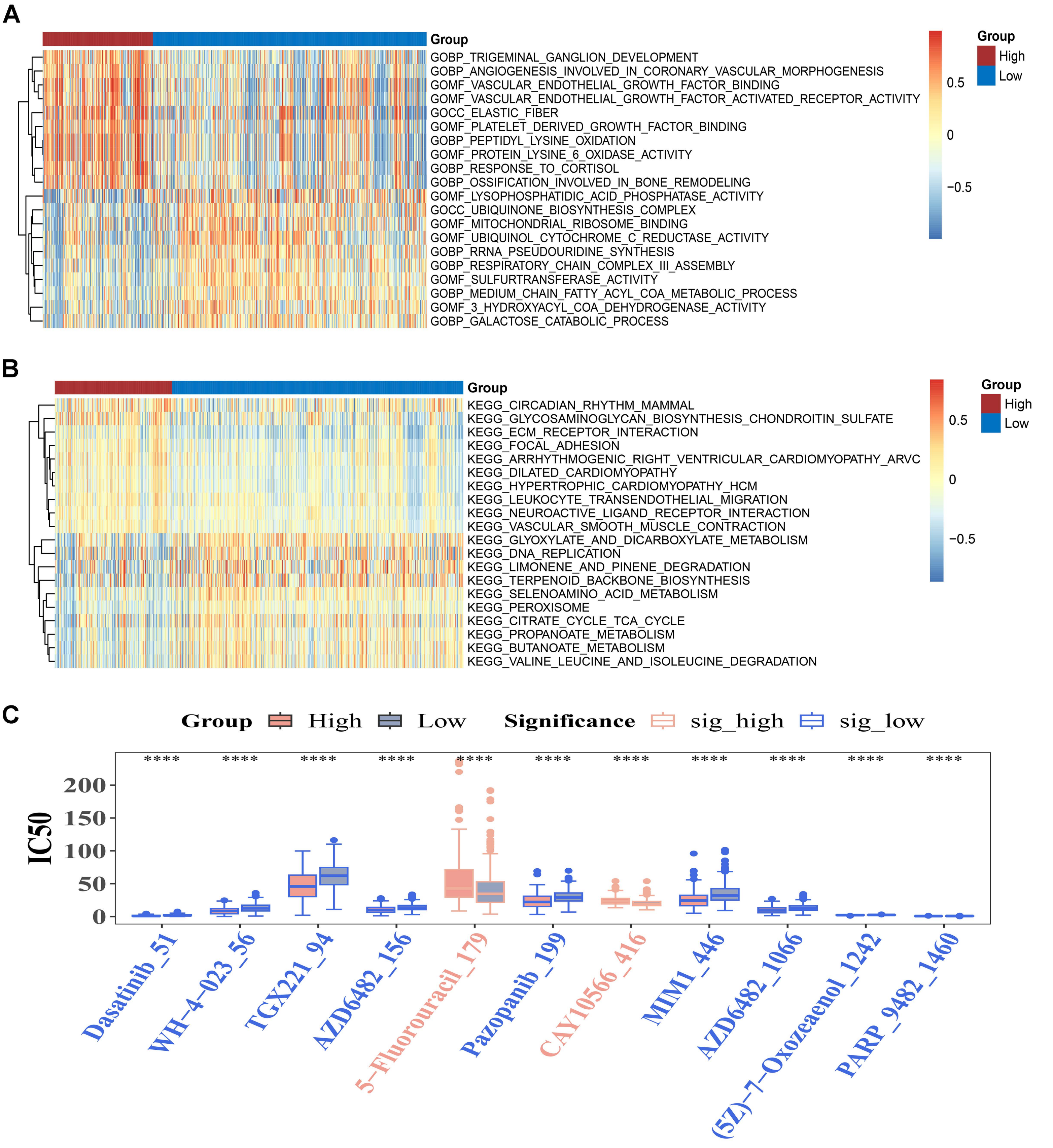
A
Group
GOBP_TRIGEMINAL_GANGLION_DEVELOPMENT
GOBP_ANGIOGENESIS_INVOLVED_IN_CORONARY_VASCULAR_MORPHOGENESIS
GOMF_VASCULAR_ENDOTHELIAL_GROWTH_FACTOR_BINDING
GOMF_VASCULAR_ENDOTHELIAL_GROWTH_FACTOR_ACTIVATED_RECEPTOR_ACTIVITY
GOCC_ELASTIC_FIBER
GOMF_PLATELET_DERIVED_GROWTH_FACTOR_BINDING
GOBP_PEPTIDYL_LYSINE_OXIDATION
GOMF_PROTEIN_LYSINE_6_OXIDASE_ACTIVITY
GOBP_RESPONSE_TO_CORTISOL
GOBP_OSSIFICATION_INVOLVED_IN_BONE_REMODELING
GOMF_LYSOPHOSPHATIDIC_ACID_PHOSPHATASE_ACTIVITY
GOCC_UBIQUINONE_BIOSYNTHESIS_COMPLEX
GOMF_MITOCHONDRIAL_RIBOSOME_BINDING
GOMF_UBIQUINOL_CYTOCHROME_C_REDUCTASE_ACTIVITY
GOBP_RRNA_PSEUDOURIDINE_SYNTHESIS
GOBP_RESPIRATORY_CHAIN_COMPLEX_III_ASSEMBLY
GOMF_SULFURTRANSFERASE_ACTIVITY
GOBP_MEDIUM_CHAIN_FATTY_ACYL_COA_METABOLIC_PROCESS
GOMF_3_HYDROXYACYL_COA_DEHYDROGENASE_ACTIVITY
GOBP_GALACTOSE_CATABOLIC_PROCESS
0.5
0
−0.5
Group
High
Low
B
Group
KEGG_CIRCADIAN_RHYTHM_MAMMAL
KEGG_GLYCOSAMINOGLYCAN_BIOSYNTHESIS_CHONDROITIN_SULFATE
KEGG_ECM_RECEPTOR_INTERACTION
KEGG_FOCAL_ADHESION
KEGG_ARRHYTHMOGENIC_RIGHT_VENTRICULAR_CARDIOMYOPATHY_ARVC
KEGG_DILATED_CARDIOMYOPATHY
KEGG_HYPERTROPHIC_CARDIOMYOPATHY_HCM
KEGG_LEUKOCYTE_TRANSENDOTHELIAL_MIGRATION
KEGG_NEUROACTIVE_LIGAND_RECEPTOR_INTERACTION
KEGG_VASCULAR_SMOOTH_MUSCLE_CONTRACTION
KEGG_GLYOXYLATE_AND_DICARBOXYLATE_METABOLISM
KEGG_DNA_REPLICATION
KEGG_LIMONENE_AND_PINENE_DEGRADATION
KEGG_TERPENOID_BACKBONE_BIOSYNTHESIS
KEGG_SELENOAMINO_ACID_METABOLISM
KEGG_PEROXISOME
KEGG_CITRATE_CYCLE_TCA_CYCLE
KEGG_PROPANOATE_METABOLISM
KEGG_BUTANOATE_METABOLISM
KEGG_VALINE_LEUCINE_AND_ISOLEUCINE_DEGRADATION
0.5
0
−0.5
Group
High
Low
C
Group High Low Significance sig_high sig_low
IC50
200
150
100
50
0
****
Dasatinib_51
WH−4−023_56
TGX221_94
AZD6482_156
5−Fluorouracil_179
Pazopanib_199
CAY10566_416
MIM1_446
AZD6482_1066
(5Z)−7−Oxozeaenol_1242
PARP_9482_1460
