# Supplementary material for: Crucial role of telomere maintenance-related genes in survival prediction and subtype identification in colorectal cancer
Source: Front Mol Biosci. 2026 Aug 28;13:1728178. doi: 10.3389/fmolb.2026.1728178 (PMC13561823; doi:10.3389/fmolb.2026.1728178)
Supplement: Supplementary file 9 [file DataSheet5.pdf]

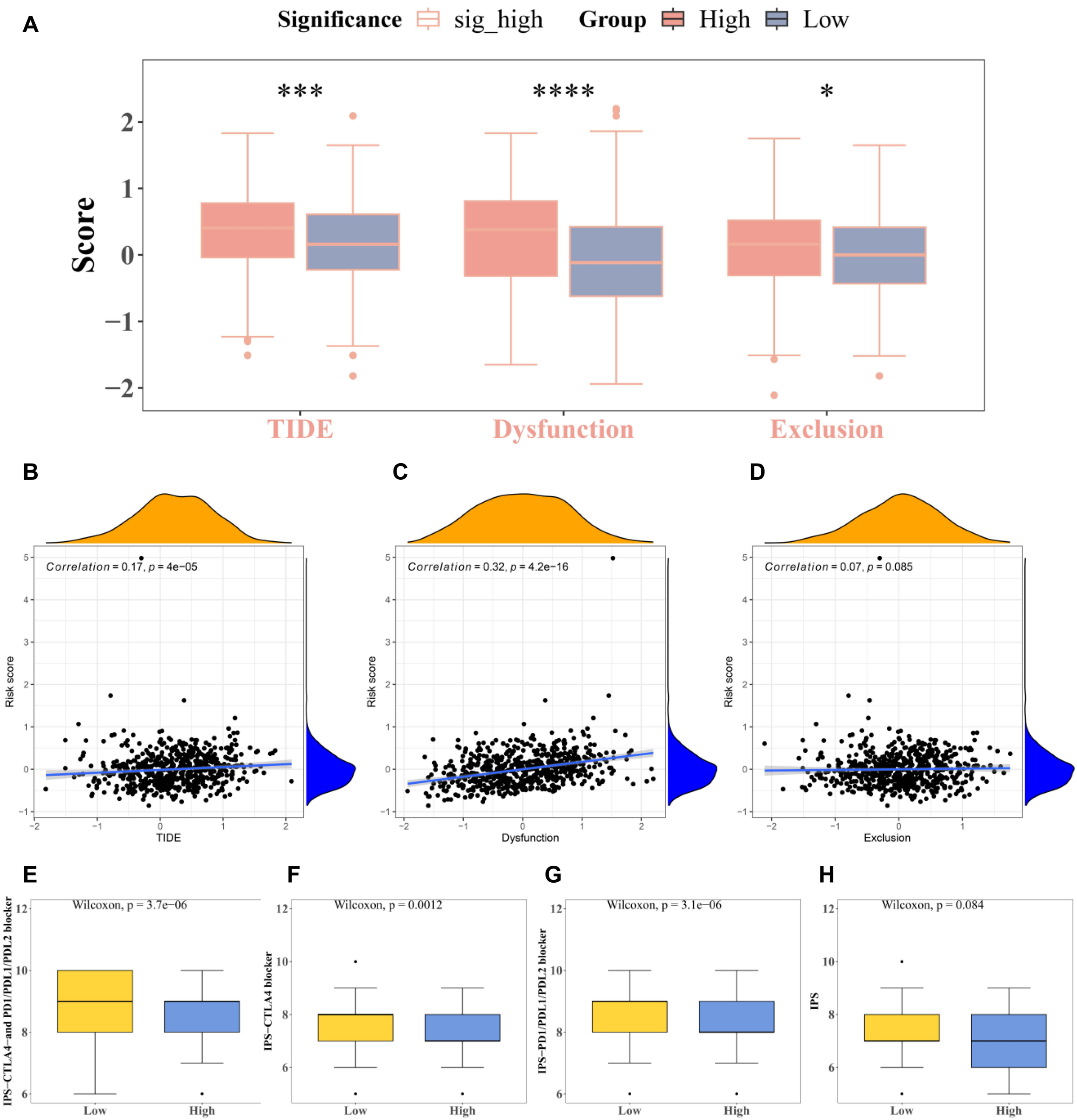
A
Significance sig_high Group High Low
***
****
*
Score
TIDE
Dysfunction
Exclusion
B
Correlation = 0.17, p = 4e−05
Risk score
TIDE
C
Correlation = 0.32, p = 4.2e−16
Risk score
Dysfunction
D
Correlation = 0.07, p = 0.085
Risk score
Exclusion
E
Wilcoxon, p = 3.7e−06
IPS−CTLA4−and PD1/PDL1/PDL2 blocker
Low
High
F
Wilcoxon, p = 0.0012
IPS−CTLA4 blocker
Low
High
G
Wilcoxon, p = 3.1e−06
IPS−PD1/PDL1/PDL2 blocker
Low
High
H
Wilcoxon, p = 0.084
IPS
Low
High
