# Supplementary material for: Crucial role of telomere maintenance-related genes in survival prediction and subtype identification in colorectal cancer
Source: Front Mol Biosci. 2026 Aug 28;13:1728178. doi: 10.3389/fmolb.2026.1728178 (PMC13561823; doi:10.3389/fmolb.2026.1728178)
Supplement: Supplementary file 10 [file DataSheet6.pdf]

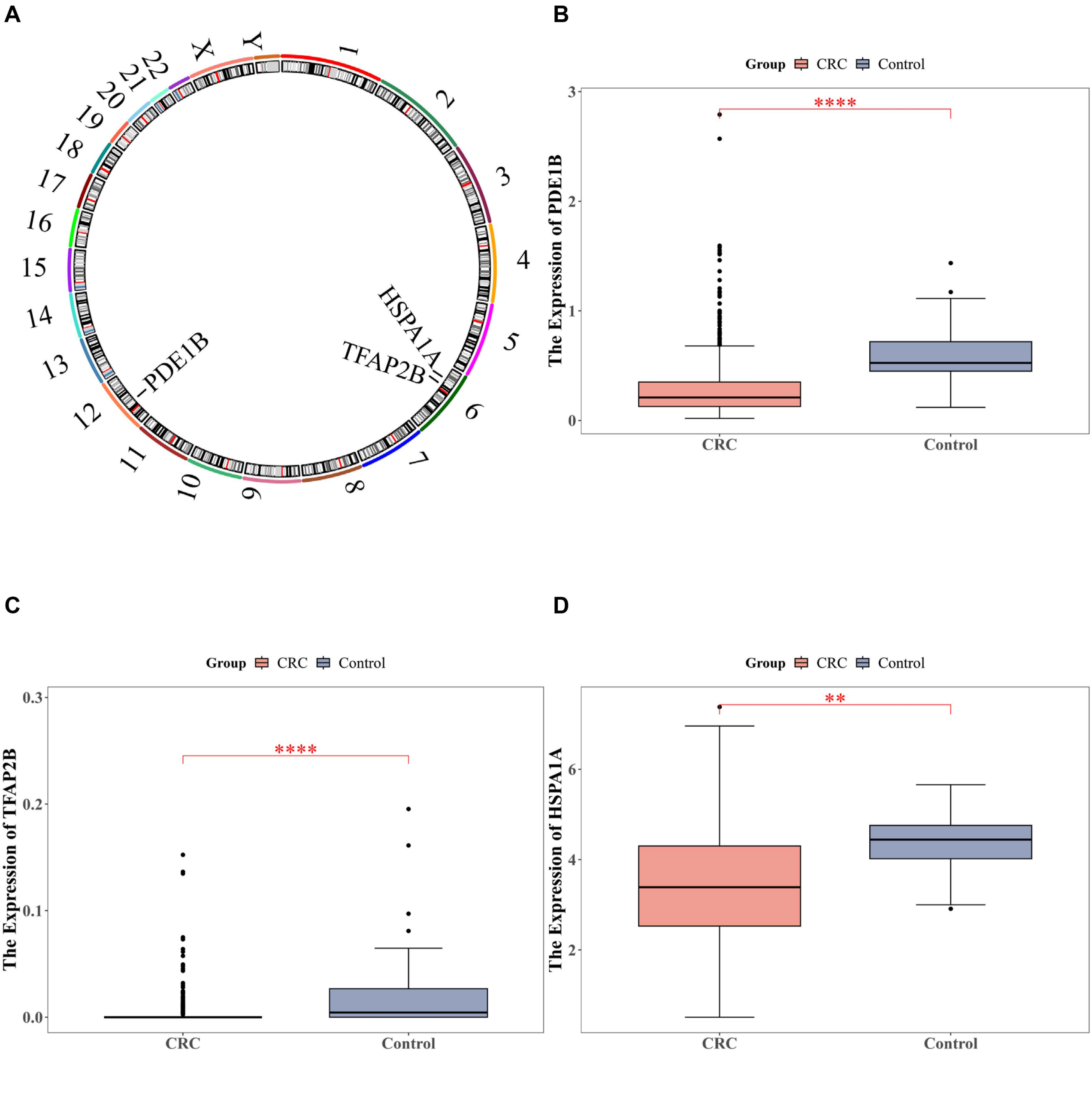

A
1
2
3
4
5
6
7
8
9
10
11
12
13
14
15
16
17
18
19
20
21
22
X
Y
HSPA1A
TFAP2B
PDE1B
B
Group CRC Control
The Expression of PDE1B
****
0
1
2
3
CRC
Control
C
Group CRC Control
The Expression of TFAP2B
****
0.0
0.1
0.2
0.3
CRC
Control
D
Group CRC Control
The Expression of HSPA1A
**
2
4
6
CRC
Control
