# Supplementary material for: Crucial role of telomere maintenance-related genes in survival prediction and subtype identification in colorectal cancer
Source: Front Mol Biosci. 2026 Aug 28;13:1728178. doi: 10.3389/fmolb.2026.1728178 (PMC13561823; doi:10.3389/fmolb.2026.1728178)
Supplement: Supplementary file 11 [file DataSheet7.pdf]

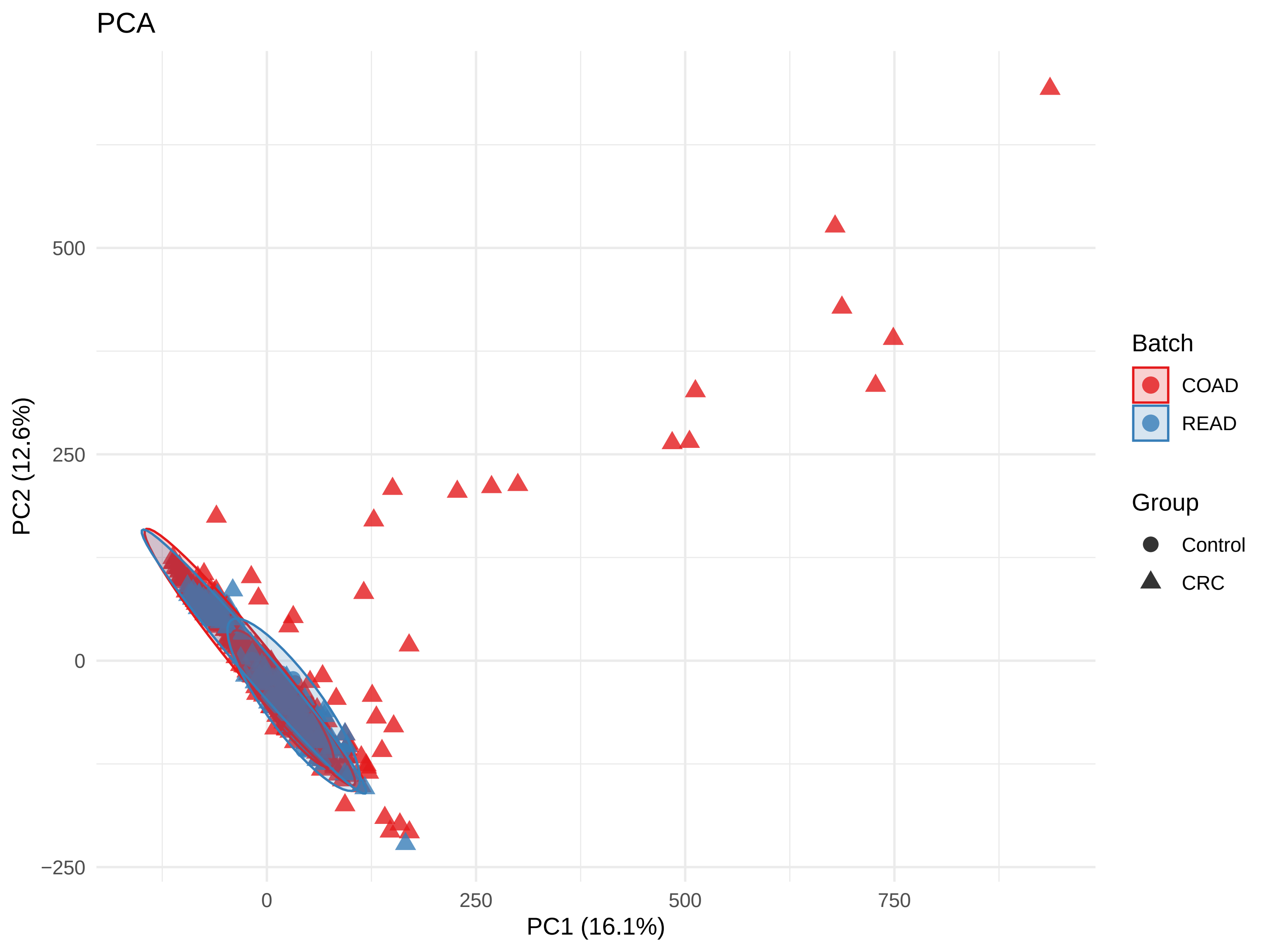
PCA
PC2 (12.6%)
PC1 (16.1%)
500
250
0
−250
0
250
500
750
Batch
COAD
READ
Group
Control
CRC
