# Supplementary material for: Crucial role of telomere maintenance-related genes in survival prediction and subtype identification in colorectal cancer
Source: Front Mol Biosci. 2026 Aug 28;13:1728178. doi: 10.3389/fmolb.2026.1728178 (PMC13561823; doi:10.3389/fmolb.2026.1728178)
Supplement: Supplementary file 12 [file DataSheet8.pdf]

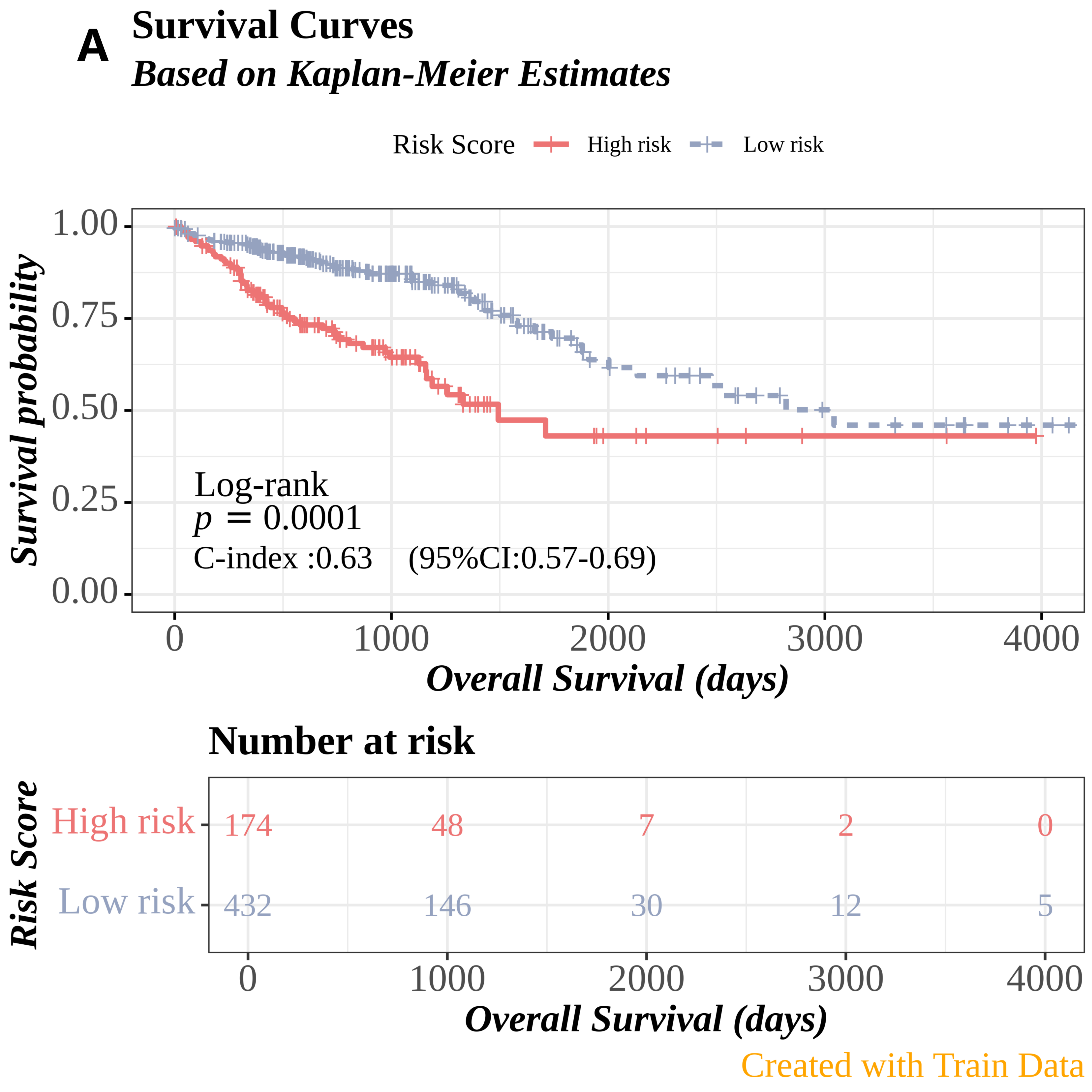
A
Survival Curves
Based on Kaplan-Meier Estimates
Risk Score
High risk
Low risk
Survival probability
1.00
0.75
0.50
0.25
0.00
Log-rank
p = 0.0001
C-index :0.63 (95%CI:0.57-0.69)
0
1000
2000
3000
4000
Overall Survival (days)
Number at risk
Risk Score
High risk
174
48
7
2
0
Low risk
432
146
30
12
5
Overall Survival (days)
Created with Train Data

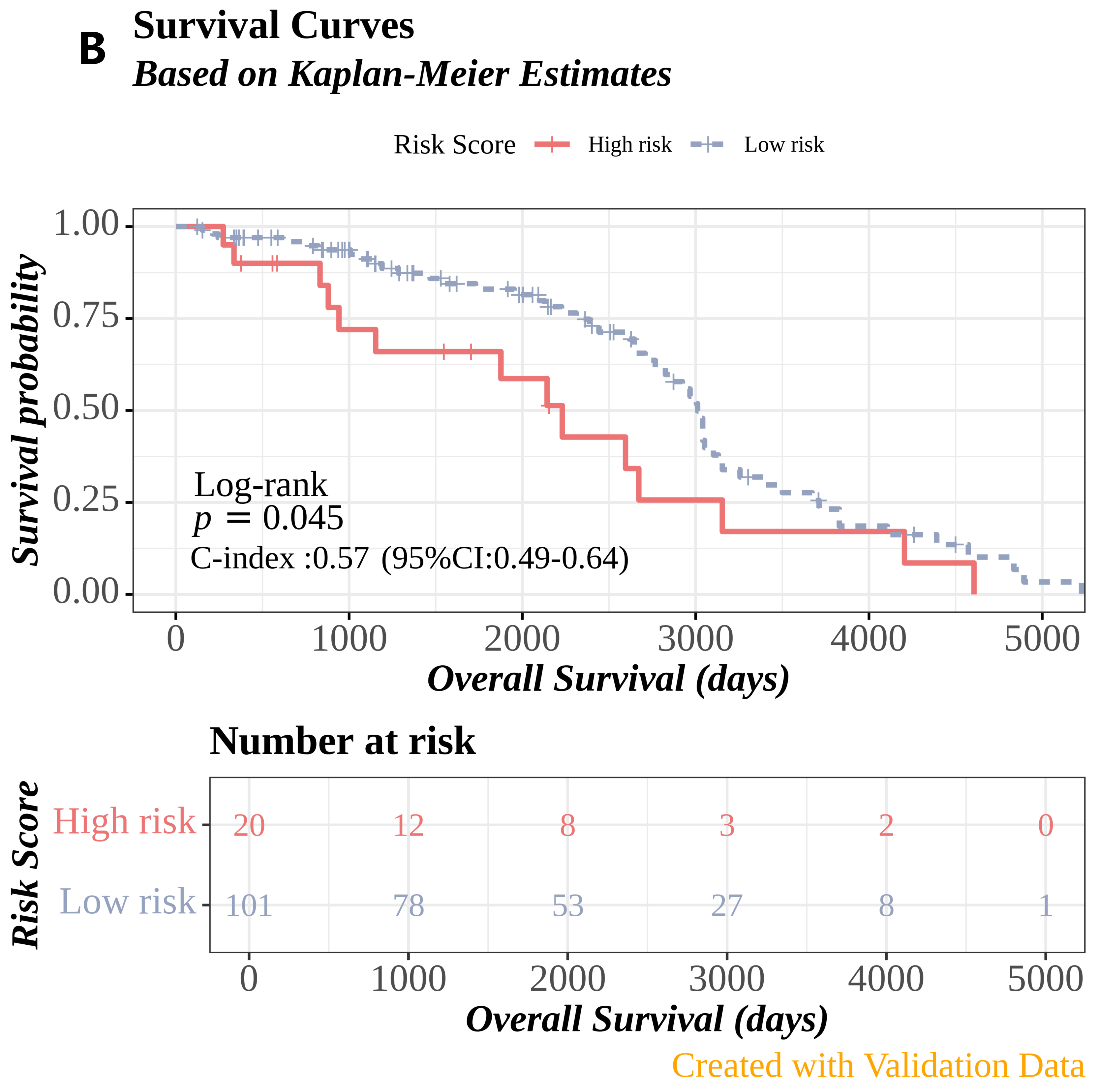
B
Survival Curves
Based on Kaplan-Meier Estimates
Risk Score
High risk
Low risk
Survival probability
1.00
0.75
0.50
0.25
0.00
Log-rank
p = 0.045
C-index :0.57 (95%CI:0.49-0.64)
0
1000
2000
3000
4000
5000
Overall Survival (days)
Number at risk
Risk Score
High risk
20
12
8
3
2
0
Low risk
101
78
53
27
8
1
Overall Survival (days)
Created with Validation Data
