# Supplementary material for: Crucial role of telomere maintenance-related genes in survival prediction and subtype identification in colorectal cancer
Source: Front Mol Biosci. 2026 Aug 28;13:1728178. doi: 10.3389/fmolb.2026.1728178 (PMC13561823; doi:10.3389/fmolb.2026.1728178)
Supplement: Supplementary file 13 [file DataSheet9.pdf]

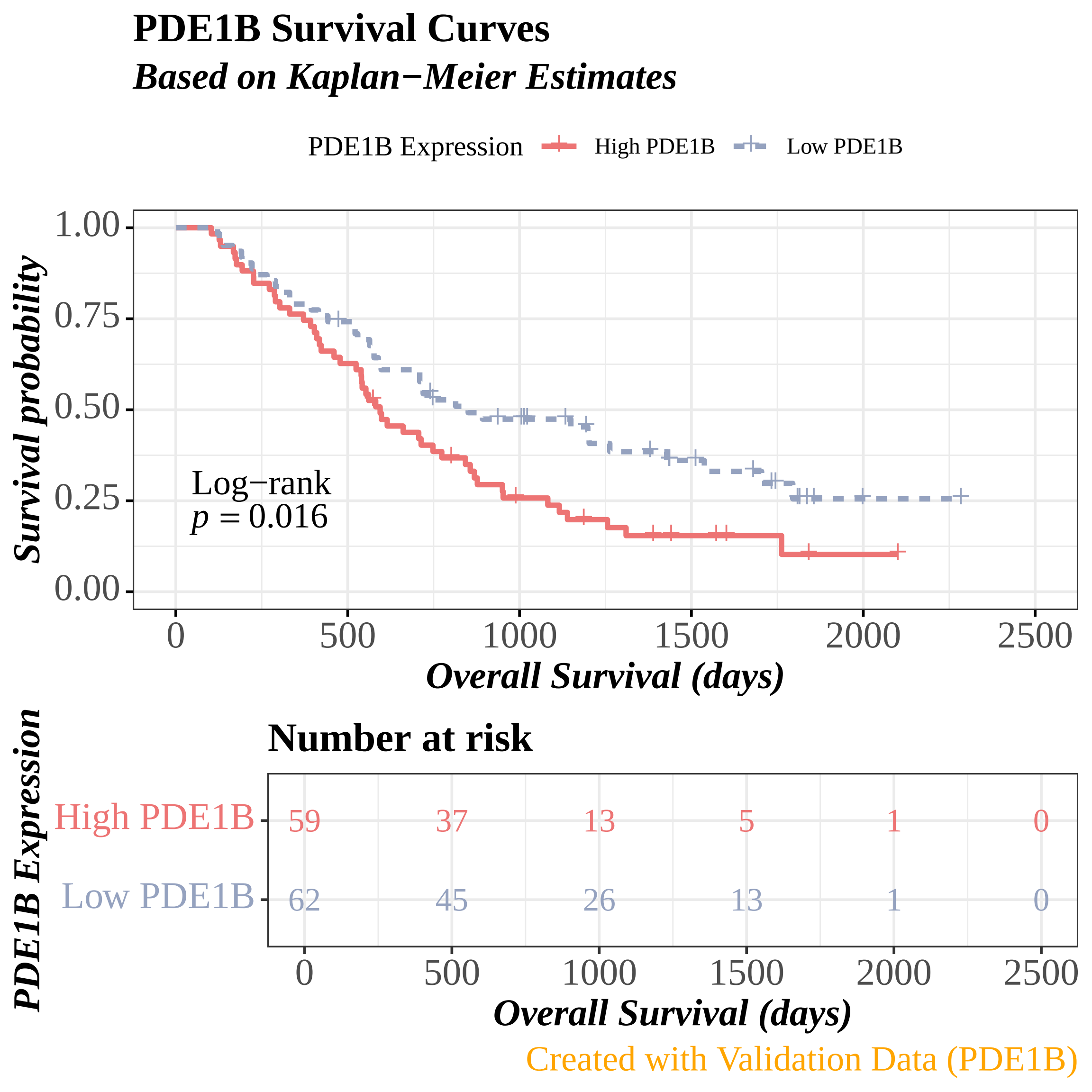
PDE1B Survival Curves
Based on Kaplan−Meier Estimates
PDE1B Expression
High PDE1B
Low PDE1B
Survival probability
1.00
0.75
0.50
0.25
0.00
Log−rank
p = 0.016
0
500
1000
1500
2000
2500
Overall Survival (days)
Number at risk
PDE1B Expression
High PDE1B 59 37 13 5 1 0
Low PDE1B 62 45 26 13 1 0
0
500
1000
1500
2000
2500
Overall Survival (days)
Created with Validation Data (PDE1B)
